# Enhanced RGB-D SLAM through orthogonal plane constraints and point-line-plane collaborative optimization

**DOI:** 10.1371/journal.pone.0330839

**Published:** 2025-09-12

**Authors:** Gaochao Yang, Pengfei Liu, Weifeng Ma

**Affiliations:** 1 College of Computer Science and Artificial Intelligence, Changzhou University, Changzhou, Jiangsu, China; 2 College of Instrumentation Science and Engineering, Southeast University, Nanjing, Jiangsu, China; 3 Department of Geography, Yunnan Normal University, Kunming, Yunnan, China; VNUHCM HCMUT: VNUHCM-Ho Chi Minh City University of Technology, VIET NAM

## Abstract

Accurate visual localization in complex indoor environments remains a significant challenge due to feature degradation and cumulative errors. In response, we propose PLPM-SLAM, a novel RGB-D SLAM framework that integrates orthogonal Manhattan plane constraints with point-line-plane joint optimization to enhance both robustness and accuracy. Unlike traditional approaches that decouple only the rotation matrix, PLPM-SLAM utilizes three mutually orthogonal planes to jointly decouple both rotation and translation, effectively mitigating global drift. To address scenarios lacking a complete Manhattan structure, we introduce a virtual plane construction strategy based on heterogeneous feature associations. Additionally, PLPM-SLAM incorporates both homogeneous (point-point, line-line, plane-plane) and heterogeneous (point-line, point-plane, line-plane) geometric constraints throughout the tracking and optimization processes. In unstructured environments, a vanishing-point–guided joint optimization model is employed to improve geometric consistency. Extensive evaluations on public datasets (TUM, ICL-NUIM) and real-world sequences demonstrate that PLPM-SLAM consistently outperforms ORB-SLAM3 in both structured and low-texture settings. Specifically, PLPM-SLAM achieves RMSE reductions of up to 82.77% and 92.16% on the public and real-world datasets, respectively.

## 1 Introduction

Robot intelligence technologies are advancing rapidly worldwide, with autonomous navigation serving as a fundamental prerequisite for realizing truly intelligent robotic systems [1,2]. In this context, Visual Simultaneous Localization and Mapping (VSLAM) plays a crucial role by enabling effective and reliable navigation. Most feature-based V-SLAM approaches rely heavily on feature points to represent the environment and estimate camera poses [3]. Owing to their computational efficiency and simple representation, feature points are widely adopted in both indoor and outdoor scenarios [4]. However, these methods face substantial challenges under complex real-world conditions, such as low-texture environments and significant lighting variations [5]. Further limitations include inaccurate data association, noise in feature point extraction, and error accumulation during pose estimation in large-scale environments, all of which hinder the robustness of SLAM systems that rely solely on feature points [6].

Indoor environments—common operational domains for mobile robots—often contain abundant structural geometric primitives such as walls, floors, and ceilings. These structures offer higher-level features, such as lines and planes, which go beyond traditional point features [7]. Compared to feature points, line and plane features are less susceptible to texture sparsity and illumination changes, enabling more consistent and reliable extraction under challenging conditions. This, in turn, enhances pose estimation accuracy and improves the overall SLAM performance, particularly in low-texture or poorly lit indoor scenarios [8,9,10]. Moreover, many man-made indoor environments exhibit dominant planar relationships such as parallelism and orthogonality. Exploiting these structural constraints allows for more robust long-term plane associations, thereby mitigating pose drift and reducing cumulative error over time [11,12].

SLAM systems rely on inter-frame pose propagation to support continuous localization and mapping, where the pose of the current frame is inferred from the previously estimated pose. Over extended operations, this propagation process leads to the accumulation of pose errors, resulting in trajectory drift and reduced mapping accuracy. The impact of such drift becomes increasingly significant in large-scale or long-duration deployments. To address these issues, two primary strategies are commonly adopted:

- Loop Closure: Detecting when the current frame resembles a previously visited scene and performing loop closure corrections to address accumulated drift. However, loop closure involves significant computational overhead and only triggers when the same scene is revisited [3].
- Manhattan World (MW) Assumption: Structured environments that adhere to the Manhattan assumption, known as MW, are rich in structural features, explicitly representing the pose relationship between the camera and world coordinates [13]. This assumption is based on the orthogonal structural patterns of buildings and indoor environments, where 3 dominant orthogonal directions exist [14].

The above review underscores that Manhattan World (MW) assumptions based solely on line features often lack robustness, particularly in environments with weak structural regularity, thereby posing significant challenges for practical applications. Traditional point-line-plane visual SLAM systems tend to process these geometric primitives independently at both the front-end and back-end stages, rarely considering their underlying spatial correlations. Moreover, most classical studies on Manhattan coordinate systems rely heavily on line features and primarily focus on decoupling rotation matrices, with limited attention given to the decoupling of translation matrices. Although many RGB-D SLAM systems incorporate point and plane features as essential observations, and several studies have successfully utilized plane features to estimate the Manhattan Frame (MF), the spatial relationships between point and plane features are often neglected.

To overcome these limitations, we propose a novel RGB-D SLAM framework, PLPM-SLAM, which jointly optimizes point, line, and plane features under Manhattan coordinate system constraints. In structured environments such as tunnels or long corridors—where typically only two orthogonal planes are observable—we introduce an auxiliary hypothetical plane construction strategy to form a complete orthogonal frame. This facilitates the decoupling of both rotation and translation matrices while preserving spatial consistency between point and plane features in the current frame. For unstructured or weakly structured environments, we further propose a joint optimization model that minimizes both homogeneous (e.g., point-to-point, plane-to-plane) and heterogeneous (e.g., point-to-plane, line-to-plane) feature association errors, guided by vanishing point constraints to enhance robustness. The key contributions of this work are summarized as follows:

(1) This study introduces PLPM-SLAM, an enhanced RGB-D SLAM framework that jointly optimizes point, line, and plane features under Manhattan plane constraints. The proposed method decouples both rotation and translation matrices to mitigate global drift commonly encountered in SLAM systems. To address scenarios where only two orthogonal planes are visible—such as in tunnels or corridors—an auxiliary hypothetical plane is constructed, enabling the formation of a complete orthogonal frame and facilitating accurate translation estimation while maintaining spatial consistency between point and plane features in the current frame.
(2) A coplanar line–assisted Agglomerative Hierarchical Clustering (AHC) strategy is developed to extract more reliable planar features. This improves the density and reliability of structural constraints, enhancing the overall stability and robustness of the system.
(3) In contrast to conventional visual SLAM methods, PLPM-SLAM integrates both homogeneous (point-to-point, line-to-line, plane-to-plane) and heterogeneous (point-to-line, point-to-plane, line-to-plane) geometric relationships during tracking and optimization. For unstructured environments, a joint optimization model guided by vanishing point constraints is proposed to simultaneously minimize geometric matching errors and semantic association inconsistencies, thereby improving robustness.
(4) The effectiveness of PLPM-SLAM is validated through extensive experiments on public benchmark datasets (TUM and ICL-NUIM) and custom datasets collected using a ZED2i RGB-D camera in real-world corridor and underground scenarios. Comparative analysis against state-of-the-art RGB-D SLAM systems—including ORB-SLAM3 [15], MSC-VO [16], Planar-SLAM [17], and Manhattan-SLAM [18]—demonstrates the superior accuracy and robustness of the proposed method.

The structure of this paper is organized as follows: the Related Work section reviews previous studies; the Manhattan Coordinate System Constraints and Nonlinear Optimization Based on Imaginary Planes section elaborates on the proposed constraints and optimization framework; the Experimental Verification and Evaluation section presents experimental results and performance analysis; finally, the Conclusion section summarizes the main findings and outlines future research directions.

## 2 Related work

The related work is presented from two perspectives, namely the line-feature-based MW and the plane-feature-based MW.

### 2.1 Manhattan world based on line features

Indoor environments—such as corners, ceilings, and other architectural structures—often conform to a globally consistent Manhattan coordinate system. Aligning the detected structural lines and plane normals in each frame with this coordinate system facilitates globally consistent rotation estimation and effectively suppresses the accumulation of pose drift [19,20]. Jiang et al. [21] proposed a general line-based SLAM framework that integrates points, structured lines, and unstructured lines. Their approach addresses the common challenges of line-only systems, such as triangulation degeneracy and unstable tracking, highlighting the importance of multi-feature fusion to improve SLAM robustness.

Lim et al. [22] and Chen et al. [23] further investigated the extraction of vanishing points using structural line features. By leveraging vanishing point constraints, they were able to reduce long-term pose drift, demonstrating the effectiveness of geometric priors in enhancing SLAM stability. Company et al. [16] introduced an RGB-D SLAM method that employs line features to constrain the Manhattan axes. Their system reduces camera pose drift through local map optimization in the backend, thereby improving overall localization accuracy and consistency. Li et al. [24] exploited the structural regularities of line features to estimate both absolute and relative camera poses. Their method introduced a novel decoupling approach for rotation and translation estimation, enabling more accurate and efficient pose inference in complex indoor scenes. Xu et al. [25] proposed a line optical flow tracking method based on grayscale invariance and collinearity constraints, significantly improving the efficiency of line-based feature matching. Wang et al. [6] developed LF-SLAM, a line-flow-based SLAM system modeled using Bayesian networks. By incorporating uncertainty and prior knowledge, their probabilistic formulation enhances the robustness of camera pose estimation. Kim et al. [26] proposed an efficient SO(3) manifold-constrained mean-shift algorithm to track spatial regularities. Their method jointly estimates drift-free rotational motion from both line and plane features, effectively leveraging environmental regularities. Straub et al. [27] presented a real-time MAP inference algorithm capable of estimating Manhattan Frames (MFs) in complex scenes. They extended the MF model to a probabilistic mixture formulation, enabling more accurate and flexible representation of real-world indoor environments.

In corridor-like environments, line-feature-based SLAM methods may fail to extract a reliable Manhattan coordinate system due to the predominance of lines aligned with the camera’s trajectory. However, such scenes typically contain two orthogonal planes (e.g., floor and wall), whose normals can be used to infer a third orthogonal direction. This allows for the construction of a complete Manhattan coordinate system, even in the absence of sufficient line features.

### 2.2 Manhattan world based on plane features

In large-scale environments, planar features tend to exhibit greater stability and continuity than line features. They are less susceptible to variations in lighting and texture and carry richer structural information [28,29]. However, the extraction and parameterization of planar features are inherently more complex compared to points and lines. Despite these challenges, numerous vision-based SLAM systems have been developed that exploit planar features [30]. For example, Zhang et al. [31] introduced SP-SLAM, which integrates point, planar, and hypothesized planar features. In their approach, planes are parameterized using spherical coordinates, and hypothesized planes are generated based on planar edge constraints and associated with contour points. Vertical and parallel geometric constraints are incorporated into the optimization process to reduce drift in indoor environments.

Li et al. [32] proposed a tightly coupled monocular visual odometry system that combines point and line features with coplanar constraints. By triangulating endpoints of lines and points, they extracted planar grids from image sequences and 3D meshes. Coplanar constraints derived from the regularity of planar structures were introduced to enhance localization accuracy. Subsequently, Li et al. [33] presented Structure-SLAM, a monocular SLAM framework that incorporates points, lines, planes, and the Manhattan World (MW) assumption. This system employs convolutional neural networks to estimate point cloud normals and uses a decoupling strategy to cluster 3D points and structural lines into dominant Manhattan directions. Given the relatively low precision of CNN-estimated normals compared to those derived from depth maps, a pose refinement module based on point and line features is integrated. Later, Li et al. [17] extended this work with Planar-SLAM, which leverages line and planar features to estimate camera pose and incorporates geometric planar relationships into bundle adjustment. However, Planar-SLAM’s performance degrades significantly in unstructured environments.

Both Planar-SLAM and Structure-SLAM employ mean-shift clustering to extract Manhattan coordinate systems. In contrast, Yunus et al. [18] proposed Manhattan-SLAM, which utilizes orthogonal plane relationships to extract the Manhattan frame. This method addresses the problem of inconsistent dominant directions in mixed Manhattan frames by detecting orthogonal plane normals and associating co-visible planes across keyframes. However, it is limited to scenes with stable planar features and fails when only parallel orthogonal planes exist between the current and historical frames. Dong et al. [34] developed an efficient Manhattan frame detection strategy based on orthogonal planes and lines, enabling decoupling of rotation and translation via the extracted Manhattan frame. UPLP-SLAM [35] introduced a unified, tightly coupled multi-feature optimization framework that employs mutual association mechanisms for both homogeneous and heterogeneous features, enhancing multimodal integration. Nonetheless, it still underutilizes structural information and relies heavily on high-precision depth data, limiting its applicability in structured environments with low-quality sensors.

Alternative models such as the Hong Kong World [36] and Atlanta World [37] have also been proposed. However, whether based on the MW, Hong Kong World, or Atlanta World assumptions, these models require the scene to conform to specific structural patterns. Other studies [38,39] have leveraged the MW assumption to estimate drift-free rotations. While these approaches yield favorable results in well-structured scenes, their performance degrades in environments lacking regular structure. Despite considerable advances, most existing Manhattan coordinate system studies rely predominantly on line features, with limited investigation into planar features. Furthermore, the majority of research focuses on the decoupling of rotation matrices, with relatively little attention given to translation matrix decoupling.

## 3 Manhattan coordinate system constraints and nonlinear optimization based on imaginary plane

In 3D space, the projection of parallel lines converging at a single vanishing point (VP) serves as a fundamental cue for constructing a Manhattan coordinate system, particularly in environments with regular architectural structures. In such scenes, three mutually perpendicular vanishing points are typically detectable, forming the basis of MF. When a scene’s structural layout is relatively regular, this coordinate system can effectively constrain global trajectory drift. Many classical vision-based Simultaneous Localization and Mapping (SLAM) approaches that adopt the Manhattan World (MW) assumption rely on line features to extract the MF. However, due to noise in line feature detection, MF estimation can become unstable and exhibit considerable fluctuations. Furthermore, these methods often assume that the principal directions of the MF remain consistent throughout the entire scene. When the dominant structural directions vary, the algorithms may fail to adapt accordingly—sometimes even leading to system crashes, as reported in [16].

**Fig 1:**
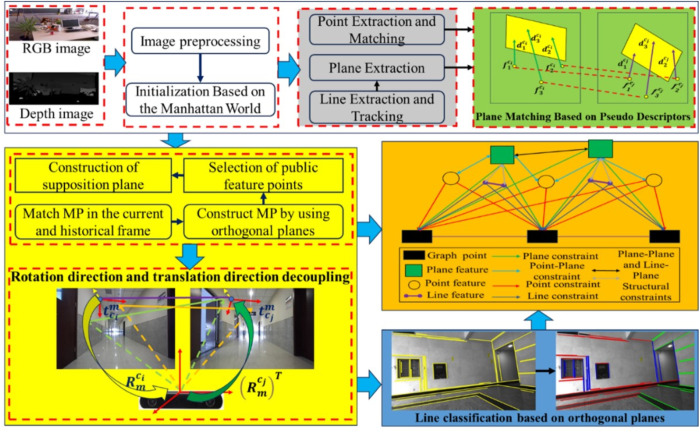
Overall architecture diagram.

Building upon the methodologies presented in [15] and [38], this paper introduces PLPM-SLAM, a novel RGB-D SLAM framework that incorporates MF system constraints through auxiliary hypothetical planes. PLPM-SLAM is designed to operate robustly in both structured and unstructured environments. Similar to the method in [38], PLPM-SLAM initially extracts the MF using detected orthogonal plane features and decouples the rotation matrix accordingly. The translation matrix is then decoupled by measuring the distances from the camera coordinate system to the three orthogonal planes. In scenarios where only two orthogonal planes are observable, the framework introduces an auxiliary hypothetical plane to complete the orthogonal triplet, thereby enabling full pose decoupling. The overall algorithmic workflow is illustrated in Fig 1.

Unlike conventional visual SLAM systems, which are typically divided into front-end and back-end modules, the proposed framework employs a three-tier architecture comprising front-end, middle-end, and back-end modules. The front-end is responsible for extracting and matching point, line, and plane features. The middle-end handles alignment with the Manhattan coordinate system and decouples the pose matrix. The back-end performs local nonlinear optimization and loop closure detection. Detailed descriptions of Manhattan plane feature matching, pose matrix decoupling, and back-end optimization are provided in “Manhattan coordinate system constraints and nonlinear optimization based on imaginary plane” section. Since loop closure is not the primary focus of this work, further analysis of that module is omitted.

As shown in Fig 1, the green module represents the plane feature tracking and matching strategy based on plane descriptors. The yellow module denotes the pose matrix decoupling process using orthogonal Manhattan plane features, including the construction of auxiliary hypothetical planes and line feature classification based on vanishing points. The brown module corresponds to the graph optimization backend, which integrates point-line-plane collaborative optimization with vanishing point constraints.

Initially, the environmental map is constructed to obtain the pose and depth information of 3D landmarks. Depth data is directly obtained through back-projection from the RGB-D camera. When orthogonal plane features are detected, they are used to extract the MF for the current frame, which serves as the initial system matrix. Subsequently, feature matching is employed for pose tracking, including tracking between adjacent frames and local maps using both homogeneous and heterogeneous features. Once the Manhattan coordinate system is successfully extracted, it is used to assist in the classification and tracking of plane and line features.

### 3.1 Association strategy between heterogeneous features of points, lines and planes

#### 3.1.1 A plane feature construction method for the AHC method assisted by coplanar line features.

Unlike points or lines, an ideal infinite plane does not have a direct projection in the image plane and must be reconstructed from 3D data. A common approach is to use the RANSAC algorithm on point clouds. However, random sampling can be problematic when the point cloud is sparse or unevenly distributed, which is often the case for feature-based landmarks. Even in well-developed SLAM systems, reconstructed sparse 3D points can only provide a rough representation of the scene structure. In contrast, human perception can interpret the scene by leveraging prior knowledge from the input images—a capability we aim to emulate.

Dirk et al. [40] introduced a foundational method for plane segmentation from point clouds using clustering algorithms. Building on this, AHCP [41] improves plane extraction accuracy by employing the AHC method. AHCP identifies point cloud blocks with the smallest Mean Squared Error (MSE) and merges them with adjacent blocks. Merged blocks that exceed a predefined error threshold are discarded.

However, the AHC method is computationally intensive. To reduce processing time, block sizes are typically set such that only larger blocks are considered for plane feature extraction. Incorporating smaller plane features into front-end tracking and back-end optimization could significantly enhance pose estimation accuracy. For RGB-D cameras, depth information becomes unreliable beyond approximately 7 meters or in the presence of transparent objects, rendering the AHC method ineffective for plane feature construction in such cases. To address these limitations and improve SLAM system stability, this study proposes a strategy that leverages coplanar line features to assist the AHC method in constructing plane features. Specifically, only coplanar line features are used for plane construction. To ensure robustness, the coplanarity of line pairs is verified before plane extraction. Plane features are parameterized using the Hesse form, which represents a plane in the world coordinate system by its unit normal vector n and the perpendicular distance d from the origin.

Eq. (1) represents line feature $L_{1}$, and Eq. (2) represents line feature $L_{2}$:


$$\frac{x-x_{1}}{m_{1}}=\frac{y-y_{1}}{n_{1}}=\frac{z-z_{1}}{p_{1}}$$ (1)

$$\frac{x-x_{2}}{m_{2}}=\frac{y-y_{2}}{n_{2}}=\frac{z-z_{2}}{p_{2}}$$ (2)

$$\left\|\begin{array}{ccc} x_{2}-x_{1} & z_{2}-z_{1} & z_{2}-z_{1}\\ m_{1} & n_{1} & p_{1}\\ m_{2} & n_{2} & p_{2} \end{array}\right\|\leq \delta$$ (3)

When the line features and the line features satisfy Eq. (3), the line features and the line features are coplanar.

Two intersecting 3D lines $L_{1}$ and $L_{2}$ can determine a plane. According to literature [42], the normal vector of the plane feature is obtained by using the cross product of $L_{1}$ and $L_{2}$ and normalization. $n_{P_{12}}$ can be obtained by Eq. (4):


$$n_{P_{12}}=\frac{L_{1}\times L_{2}}{\left\|L_{1}\times L_{2}\right\|}$$ (4)

The distance $d_{P_{12}}$ can be computed using the following formula, as described in [17]:


$$d_{P_{1,2}}=\frac{\left(d_{1,s}+d_{1,e}+d_{2,s}+d_{2,e}\right)\times n_{P_{1,2}}}{4}$$ (5)

where, $d_{1,s}$ and $d_{1,e}$ are the coordinates of the two endpoints of line $L_{1}$; $d_{2,s}$ and $d_{2,e}$ are the coordinates of the two endpoints of line $L_{2}$. To further eliminate the non-planar line features, we calculate the maximum value $d_{max}$ and the minimum value $d_{min}$ of the distance respectively.



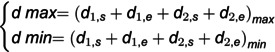

(6)


Check whether $\left|d_{max}-d_{min}\right|<\partial$ is satisfied. If not, it will be eliminated.

#### 3.1.2 A novel plane feature tracking and matching strategy based on plane feature descriptor.

This paper introduces an association strategy that leverages the mutual inclusion of both homogeneous and heterogeneous features, including point-line, point-plane, and line-plane relationships [35]. The proposed strategy considers the geometric associations among these features at both the global level—between the current frame and all features within the global map—and the local level—between the current frame and the most recent frame, as well as between the current frame and keyframes.

**Fig 2:**
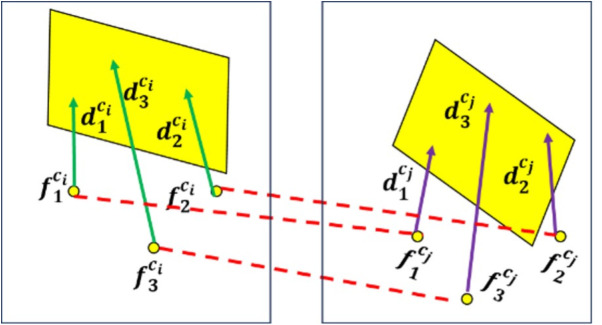
Schematic diagram of point-to-plane association strategy.

SLAM systems typically operate under the assumption of a rigid environment, wherein the relative spatial relationships among features within the same frame remain invariant. Based on the principle of rigid body motion and assuming minimal detection errors, the distances between corresponding features across different image frames should remain consistent. For instance, as illustrated in Fig 2, the distance between two point features, or between a point and a plane feature, remains unchanged across frames.

**Fig 3:**
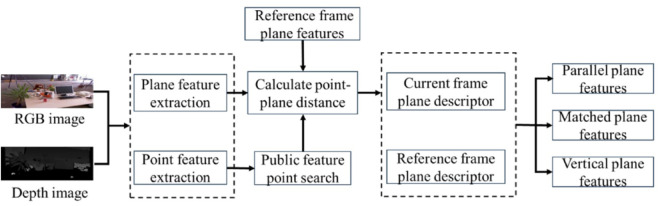
Flowchart of plane feature construction and matching.

To address the low efficiency of plane feature matching, this study introduces a data association strategy based on plane feature descriptors. The specific steps are as Fig 3 below:

First, identify *n* (where *n* ≥ 4) common keypoints between the current frame and the reference frame. Obtain the 3D coordinates of these keypoints in the respective camera coordinate systems of both frames. Next, compute the distances from each of the n keypoints to every plane feature in both frames and store these distances in a container. The distances are then sorted in descending order to construct descriptors for the corresponding plane features. Plane feature matching is subsequently performed based on these descriptors. As this matching strategy does not depend on camera pose information, it demonstrates strong robustness against pose estimation errors and enables accurate plane correspondence. Furthermore, the method supports simultaneous matching of structural plane configurations, including parallel and perpendicular planes.

### 3.2 Manhattan coordinate system constraints based on the construction of imaginary planes

**Fig 4:**
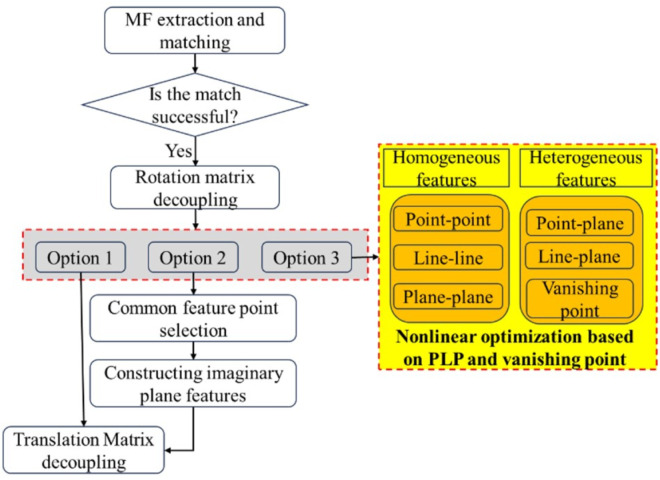
Flowchart of posture decoupling based on Manhattan coordinate system.

This paper proposes a method to decouple the rotation and translation matrices using orthogonal Manhattan plane features. Rotation matrix decoupling requires either (i) the alignment or parallelism of the normal vectors of three mutually orthogonal plane features between the current and historical frames, or (ii) the alignment or parallelism of the normal vectors of two plane features. In contrast, translation matrix decoupling necessitates exact correspondence among all three plane features. Accordingly, rotation matrix decoupling is performed first. Once the rotation matrix is successfully decoupled, the consistency of the three plane features is verified. If all three planes are consistent, translation matrix decoupling is performed. Otherwise, conventional algorithms cannot be applied for translation decoupling. The detailed process is illustrated in Fig 4.

For long corridor environments, this paper introduces a translation matrix decoupling method assisted by hypothetical plane features. When corresponding Manhattan plane features cannot be matched, pose estimation is performed using a collaborative point-line-plane optimization model constrained by vanishing points. This model jointly minimizes homogeneous feature matching errors and heterogeneous feature association errors.

#### 3.2.1 Rotation matrix decoupling based on manhattan coordinate system.

**Fig 5:**
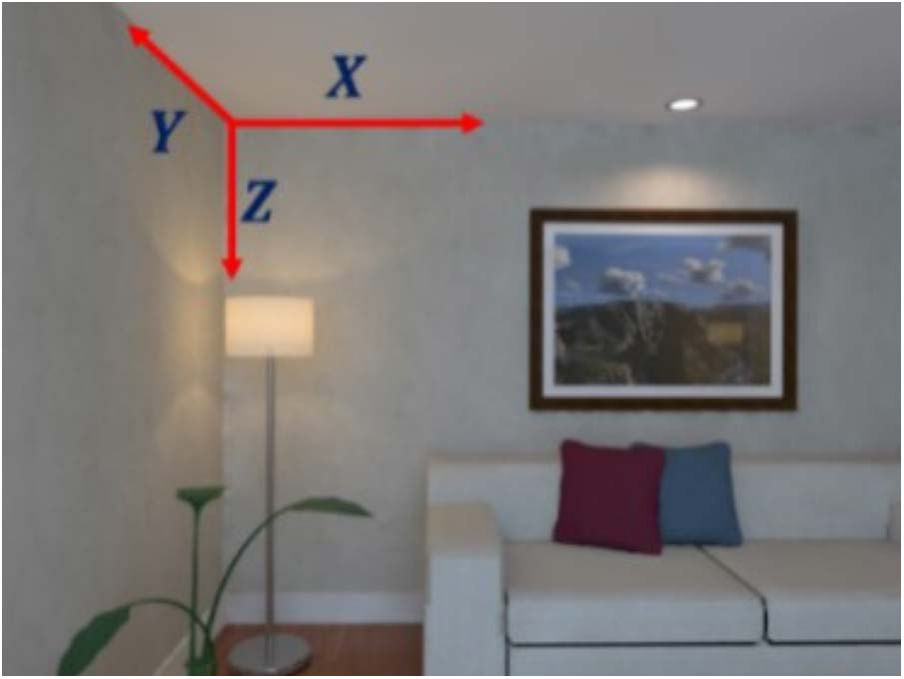
The specific definitions of MF and MP, as well as the relationship between them.

First, we introduce the following definition. As illustrated in Fig 5, suppose three mutually perpendicular lines (represented by red arrows) are detected in the image. The coordinate system formed by these three orthogonal directions is referred to as the Manhattan coordinate system, also known as the MF. The three mutually orthogonal planes that contain these lines are referred to as Manhattan planes (MPs).

Let $R_{m_{i}}^{c_{i}}$ denote the rotation matrix from the camera coordinate system to the Manhattan coordinate system in frame $c_{i}$, constructed using three orthogonal plane features extracted from the historical frame $c_{i}$. Let $R_{c_{i}}^{w}$ represent the rotation matrix from the camera coordinate system in frame $c_{i}$ to the world coordinate system. Similarly, let $R_{m_{j}}^{c_{j}}$ be the rotation matrix from the camera coordinate system to the Manhattan coordinate system in the current frame $c_{j}$, constructed using three mutually orthogonal plane features detected in $c_{j}$, which are matched with those used in $R_{c_{j}}^{w}$ —the rotation matrix from the camera coordinate system in $c_{j}$ to the world coordinate system. In this case, $R_{c_{j}}^{w}$ is the unknown quantity to be determined.

**Fig 6:**
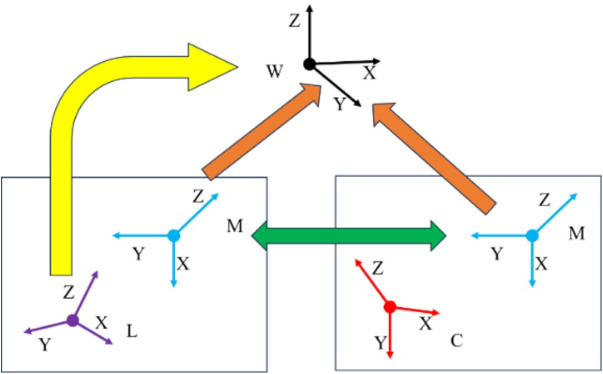
The relationship between the world coordinate system (W), the historical frame camera coordinate system (L), the Manhattan coordinate system (M), and the current frame camera coordinate system (C).


As illustrated in Fig 6, if the rotation matrix of the MF in $c_{j}$ relative to the world coordinate system can be obtained, then the camera’s rotation matrix $R_{c_{j}}^{w}$ can also be determined accordingly. Since the MF detected in both $c_{i}$ and $c_{j}$ is consistent, their respective rotation matrices to the world coordinate system must also be consistent. Given that $R_{c_{i}}^{w}$ is already known (the rotation matrix from the camera coordinate system in $c_{i}$ the world coordinate system), the rotation matrix of the MF in frame $c_{i}$ relative to the world coordinate system can be computed as in Eq. (7):

$$R_{m_{i}}^{w}=R_{c_{i}}^{w}{\left(R_{m_{c_{i}}}^{c_{i}}\right)}^{-1}$$ (7)

When the current frame $c_{j}$ detects three mutually orthogonal plane features, a MF system $m_{j}$ can be constructed according to the principles of the MW assumption. The normal vectors of these three planes define the coordinate axes of $m_{j}$, and the rotation matrix from the camera coordinate system in $c_{j}$ to the MF system is denoted as $R_{m_{j}}^{c_{j}}$. Assuming that the same set of three orthogonal plane features can also be identified in a historical frame $c_{i}$, the corresponding Manhattan coordinate system is denoted as $m_{i}$, with the rotation matrix from the camera coordinate system in $c_{i}$ to the Manhattan frame represented as $R_{m_{i}}^{c_{i}}$. Since the Manhattan frames $m_{j}$ and $m_{i}$ are consistent, the rotation matrix of the camera in frame $c_{j}$ with respect to the world coordinate system can be inferred. Following the approach in [18], the relationship can be expressed by the Eq. (8):


$$R_{c_{j}}^{w}=R_{m_{j}}^{w}{\left(R_{m_{j}}^{c_{j}}\right)}^{-1}=R_{m_{i}}^{w}{\left(R_{m_{j}}^{c_{j}}\right)}^{-1}=R_{c_{i}}^{w}{\left(R_{m_{c_{i}}}^{c_{i}}\right)}^{-1}{\left(R_{m_{j}}^{c_{j}}\right)}^{-1}$$ (8)

From Eq. (8), it can be seen that since $R_{m_{j}}^{c_{j}}$ and $R_{m_{i}}^{c_{i}}$ depend solely on the accuracy of plane feature extraction within their respective frames, their computation does not involve error accumulation across frames. Therefore, the accuracy of $R_{c_{j}}^{w}$ is directly influenced only by the accuracy of $R_{c_{i}}^{w}$, and is unaffected by errors in other intermediate frames. In other words, assuming that the estimation errors of $R_{m_{j}}^{c_{j}}$ and $R_{m_{i}}^{c_{i}}$ are negligible, the accuracy of $R_{c_{j}}^{w}$ can be considered equivalent to that of $R_{c_{i}}^{w}$.

**Fig 7:**
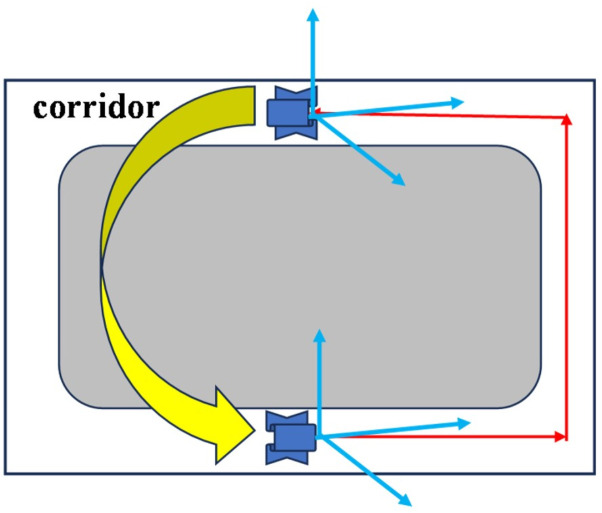
Two parallel orthogonal plane features.



- In scenarios where only two mutually orthogonal plane features or parallel orthogonal planes can be detected and matched in the scene:

When frame $c_{j}$ detects only two mutually orthogonal plane features, as illustrated in Fig 7, the cross product of the two normal vectors can be used to infer the third orthogonal direction, thereby completing the MF. Alternatively, when frame $c_{j}$ cannot detect the same orthogonal planes as in the historical frame but can detect parallel orthogonal planes, the system can still extract the normal vectors of these parallel orthogonal planes. These vectors can be used to reconstruct a Manhattan coordinate system and enable rotation matrix decoupling, as detailed in the following steps:

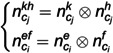

(9)


Let $n_{c_{j}}^{k}$ and $n_{c_{j}}^{h}$ denote the normal vectors of the two mutually orthogonal plane features detected in frame $c_{j}$. The third orthogonal direction $n_{c_{j}}^{kh}$ is obtained via the cross product of $n_{c_{j}}^{k}$ and $n_{c_{j}}^{h}$. Similarly, let $n_{c_{i}}^{e}$ and $n_{c_{i}}^{f}$ represent the normal vectors of two orthogonal plane features detected in frame $c_{i}$, and the vector $n_{c_{i}}^{ef}$ is the third direction obtained from the cross product of $n_{c_{i}}^{e}$ and $n_{c_{i}}^{f}$. Based on these orthogonal vectors, the rotation matrices of the MF relative to the camera coordinate system in frames $c_{i}$ and $c_{j}$ can be constructed as shown in Eq. (10):



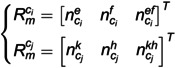

(10)

**Fig 8:**
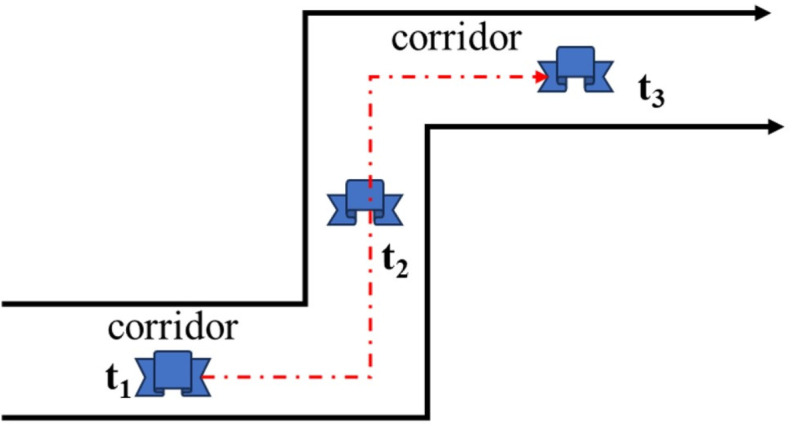
One parallel or orthogonal plane feature and another orthogonal vertical plane feature.



- When only one pair of parallel or orthogonal plane features and another orthogonal vertical plane feature can be detected and matched in the scene:

As illustrated in Fig 8, the third orthogonal direction can still be determined using the cross product of the available plane normals, following the same procedure as described previously. Then, the rotation matrices are constructed. For instance, let $n_{c_{i}}^{e}$ be a normal vector that is either parallel or orthogonal to $n_{c_{j}}^{k}$, and let $n_{c_{i}}^{f}$ be orthogonal to $n_{c_{j}}^{h}$. If the resulting MF systems $m_{i}$ and $m_{j}$ are not equivalent (i.e., $m_{i}\neq m_{j}$), the corresponding rotation matrices can be constructed as follows:

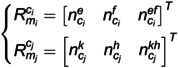

(11)


For example, when the camera detects only the ground plane feature and the wall plane feature from time $t_{1}$ to $t_{2}$, we can apply the right-hand rule and define the matrix E as: $E=[\begin{array}{ccc} \begin{array}{c} 1\\ 0\\ 0 \end{array} & \begin{array}{c} 0\\ 0\\ -1 \end{array} & \begin{array}{c} 0\\ 1\\ 0 \end{array} \end{array}]$.

This matrix is used to rotate $m_{j}$ to align with $m_{i}$. After unifying the indices, the rotation matrix is given by:


$$R_{m}^{c_{j}}=E\times R_{m_{j}}^{c_{j}}$$ (12)

#### 3.2.2 Translation matrix decoupling based on manhattan coordinate system.

**Fig 9:**
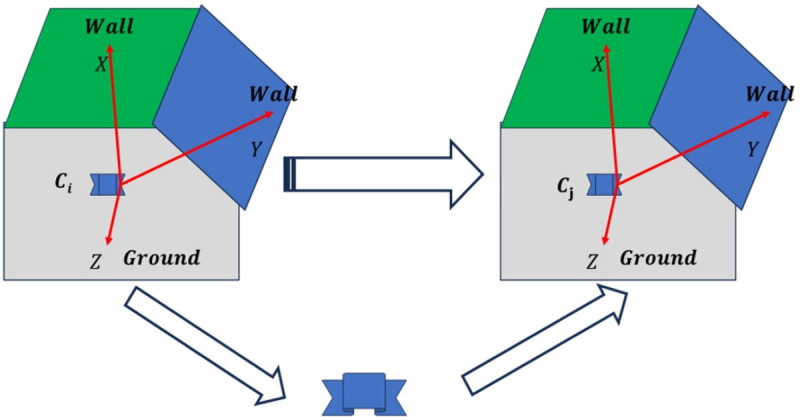
Decoupling of the translation matrix.

As shown in Fig 9, once the rotation matrix is decoupled, the decoupling of the translation matrix can typically be classified into three scenarios:

(1) Matching three fully orthogonal plane features;

Let $t_{c_{i}}^{w}$ and $R_{c_{i}}^{w}$ denote the translation and rotation matrices from the camera coordinate system of frame $c_{i}$ to the world coordinate system, respectively. It is assumed that $R_{c_{i}}^{w}$ has already been decoupled via the MF system. The vector $t_{c_{i}}^{m}=[\begin{array}{ccc} X_{i} & Y_{i} & Z_{i} \end{array}]T$ represents the distances from the origin of the camera coordinate system in frame ${\;c}_{i}$ to the MF system constructed using the MP features. The rotation matrix from the MF system (constructed in frame $c_{i}$) to the world coordinate system is denoted as ${\;R}_{m}^{w}$, which can be obtained using $t_{c_{i}}^{m}$, $t_{c_{i}}^{w}$ and ${\;R}_{c_{i}}^{w}$, as shown in Eq. (13).


$$R_{m}^{w}=R_{c_{i}}^{w}\times R_{m}^{c_{i}}$$ (13)

Assuming that frame $c_{j}$ observes the same set of three mutually orthogonal plane features, let $t_{c_{j}}^{m}=[\begin{array}{ccc} X_{j} & Y_{j} & Z_{j} \end{array}]T$ represent the distances from the origin of the camera coordinate system in frame $c_{j}$ to its corresponding MF system. The rotation matrix $R_{c_{j}}^{w}$ from frame $c_{j}$’s camera coordinate system to the world coordinate system is assumed to have already been obtained through MF decoupling. To compute the translation matrix $t_{c_{j}}^{w}$, we first estimate the translation matrix $t_{m_{i}}^{w}$, which denotes the translation from the MF system constructed in frame $c_{i}$ to the world coordinate system.


$$t_{m}^{w}=t_{c_{i}}^{w}-R_{m}^{w}\times t_{c_{i}}^{m}$$ (14)

Given that $R_{m_{i}}^{w}=R_{m_{j}}^{w}$, and $t_{m_{i}}^{w}=t_{m_{j}}^{w}$, we define $t_{m}^{w}$ as the translation matrix from the Manhattan coordinate system to the world coordinate system, and $R_{m}^{w}$ as the corresponding rotation matrix. Once $t_{m}^{w}$ is computed, the translation matrix $t_{c_{j}}^{w}$ can be obtained based on the intrinsic relationships among the world coordinate system, MF system, and camera coordinate system:


$$t_{c_{j}}^{w}=t_{m}^{w}+R_{m}^{w}\times t_{c_{j}}^{m}$$ (15)

**Fig 10:**
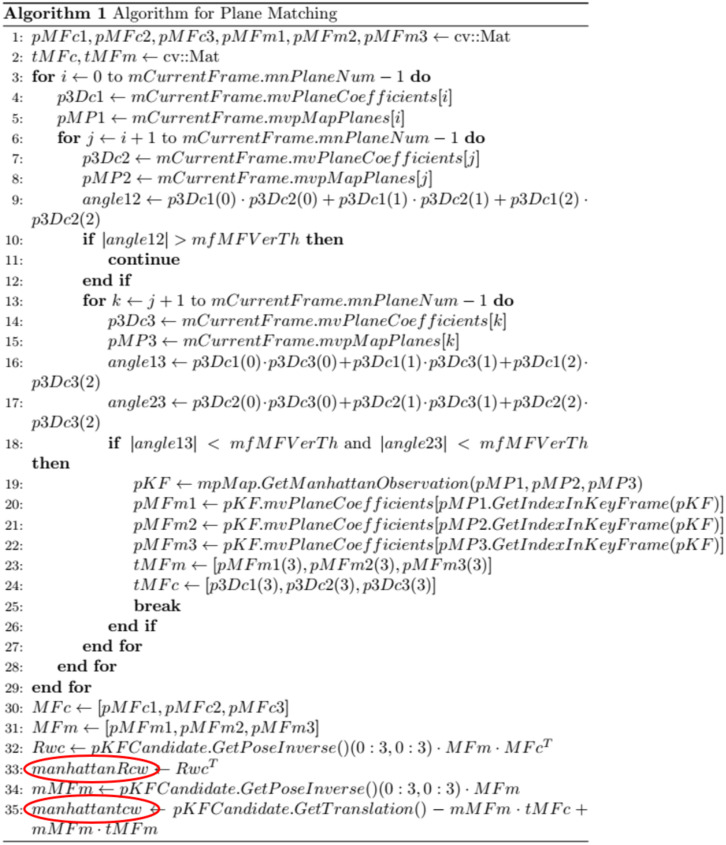
Pseudocode implementation of pose matrix decoupling.

From Eq. (15), it is evident that the translation matrix $t_{c_{j}}^{w}$ depends solely on $t_{m}^{w}$ and $R_{m}^{w}$, under the assumption that there is no error in $t_{c_{j}}^{m}$, $R_{m}^{c_{i}}$ and $t_{c_{i}}^{m}$. Since $t_{m}^{w}$ itself is only dependent on $R_{c_{i}}^{w}$, the final translation matrix $t_{c_{j}}^{w}$ is thus determined exclusively by the pose of the camera in frame $c_{i}$, remaining independent of poses in other frames. Therefore, provided that $t_{m}^{w}$ and $R_{c_{i}}^{w}$ are initialized without error accumulation, the translation matrix $t_{c_{j}}^{w}$ can be accurately decoupled from the camera coordinate system in frame $c_{j}$ to the world coordinate system. Crucially, this process is unaffected by any intermediate pose errors between frames. Fig 10 presents the pseudocode for the pose decoupling procedure, where the sections highlighted in red indicate the decoupled translation matrix components.

For simplification, when the MF system constructed from the three-plane features is aligned with the initial world coordinate system, the translation matrix $t_{c_{j}}^{w}$ can be directly simplified as $t_{c_{j}}^{m}$.

(2) Matching two completely orthogonal plane features;

Assume that only two mutually orthogonal plane features are detected in frame $c_{j}$, and the same two orthogonal planes are also detected in frame $c_{i}$. However, the MF system established during initialization is inconsistent with the one currently detected—meaning that the Manhattan coordinate system in the current frame is not aligned with the world coordinate system. In this scenario, direct translation matrix decoupling becomes infeasible.

**Fig 11:**
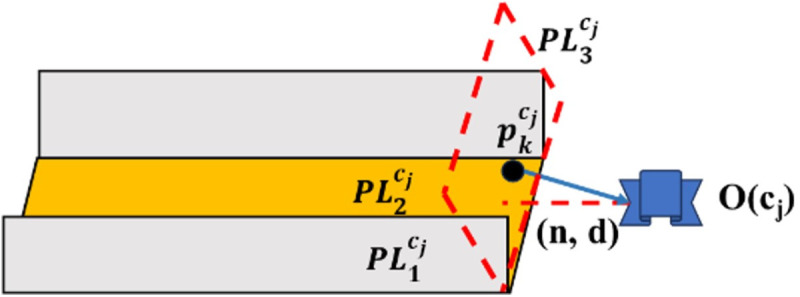
Constructing imaginary plane features using common points in frame.

**Fig 12:**
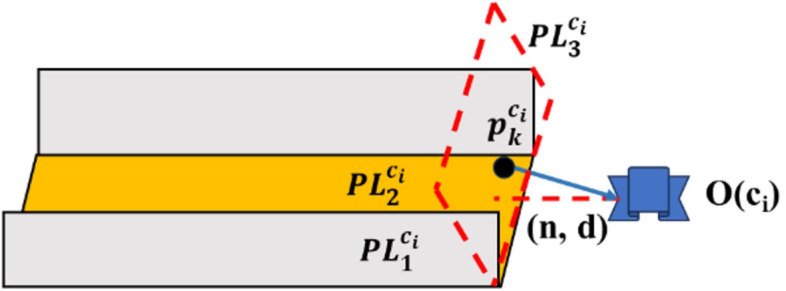
Constructing imaginary plane features using common points in frame.

To resolve this issue, we propose a translation matrix decoupling method assisted by a hypothetical plane feature. As illustrated in Figs 11 and 12, the algorithm traverses frames $c_{i}$ and $c_{j}$ to identify point features that are jointly observed. Assume that m such point features are observed by both frames. Among these, we select the most stable point feature, denoted as $p_{k}^{w}$, based on the following criteria: $p_{k}^{w}$ is observed in the largest number of keyframes, and the distance from $p_{k}^{w}$ to the camera center is less than 7 meters. The coordinates of this point feature in the camera coordinate system of frame $c_{i}$ are denoted as $p_{k}^{c_{i}}$, and in frame $c_{j}$ as $p_{k}^{c_{j}}$.

Here, $PL_{1}^{c_{j}}$ and $PL_{2}^{c_{j}}$ are a pair of orthogonal plane features detected in frame $c_{j}$; $PL_{1}^{c_{i}}$ and $PL_{2}^{c_{i}}$ are a pair of orthogonal plane features detected in frame $c_{i}$ that match $PL_{1}^{c_{j}}$ and $PL_{2}^{c_{j}}$; $PL_{3}^{c_{j}}$ is the hypothetical plane feature that needs to be constructed in frame $c_{j}$ to be orthogonal to $PL_{1}^{c_{j}}$ and $PL_{2}^{c_{j}}$; $PL_{3}^{c_{i}}$ is the hypothetical plane feature that needs to be constructed in frame $c_{i}$ to be orthogonal to $PL_{1}^{c_{i}}$ and $PL_{2}^{c_{i}}$. The Hesse representation of $PL_{3}^{c_{j}}$ can be expressed as:


$$PL_{3}^{c_{j}}=\left(n_{3}^{c_{j}},p_{c_{j}}^{k}\frac{n_{3}^{c_{j}}}{\left\|n_{3}^{c_{j}}\right\|}\right)$$ (16)

where $n_{3}^{c_{j}}=n_{1}^{c_{j}}⊗n_{2}^{c_{j}}$; $n_{1}^{c_{j}}$ and $n_{2}^{c_{j}}$ are the normal vectors of the plane features $PL_{1}^{c_{j}}$ and $PL_{2}^{c_{j}}$, respectively.

The Hesse representation of $PL_{3}^{c_{i}}$ can be expressed as:


$$PL_{3}^{c_{i}}=\left(n_{3}^{c_{i}},p_{c_{i}}^{k}\frac{n_{3}^{c_{i}}}{\left\|n_{3}^{c_{i}}\right\|}\right)$$ (17)

where $n_{3}^{c_{i}}=n_{1}^{c_{i}}⊗n_{2}^{c_{i}}$; $n_{1}^{c_{i}}$ and $n_{2}^{c_{i}}$ are the normal vectors of the plane features $PL_{1}^{c_{i}}$ and $PL_{2}^{c_{i}}$, respectively.

After constructing the hypothetical plane feature, the three orthogonal plane features from the historical and current frames are completely matched. Therefore, the translation matrix can be decoupled using the scheme in step (1).

(3) Matching orthogonal plane features that are parallel to each other.

For example, when only parallel orthogonal plane features can be matched, it becomes impossible to determine the distance relationship between the camera optical center and the three orthogonal plane features in the current and historical frames. As a result, only the rotation matrix can be decoupled, while the translation matrix cannot. In this case, the translation matrix is solved using nonlinear optimization with the cooperation of points, lines, and planes.

### 3.3 Pose estimation model based on joint optimization of points, lines and planes and vanishing point constraints

**Fig 13:**
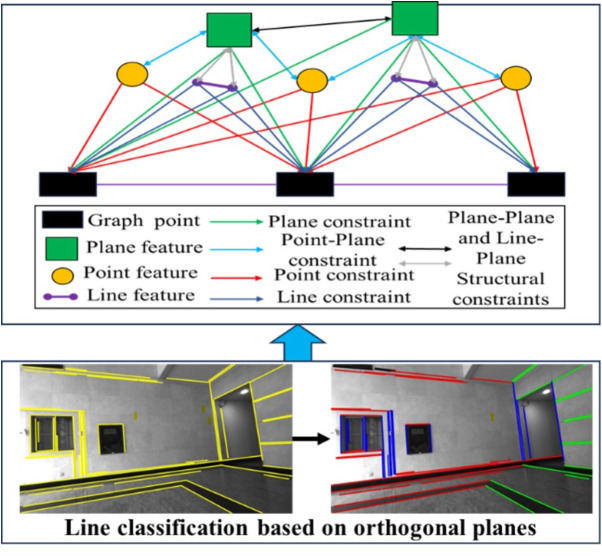
Pose estimation model based on joint optimization of points, lines and planes and vanishing point constraints.

After local map tracking, when more than two mutually orthogonal planes are detected and matched, the rotation matrix is first decoupled, followed by decoupling of the translation matrix using the hypothetical plane-assisted method. In cases where only parallel orthogonal planes are detected and matched, the rotation matrix is still decoupled first, and then nonlinear optimization is applied to the current frame using point-plane feature constraints and structural relationships to solve for the translation matrix. If fewer than two mutually orthogonal planes can be detected and matched, the system directly employs point-line-plane features and structural constraints for local nonlinear pose optimization to jointly estimate both the rotation and translation matrices, as illustrated in Fig 13.

This paper proposes a joint optimization model based on point, line, and plane (PLP) features, which simultaneously minimizes both the matching errors of homogeneous features and the association errors of heterogeneous features. To enhance the accuracy of multi-feature SLAM systems and better utilize the strengths of line and plane features during tracking, we adopt a unified parameter representation using point features for all feature types during parameter estimation and nonlinear optimization. Following the method in [43], line features are represented by the start endpoint of the line segment, while plane features are represented by two coplanar line segments. Referencing [15] and [18], all state variables are optimized by minimizing the total cost from all measurement residuals, as expressed in Eq. (18):


$$\begin{array}{c} min\sum_{j\in F}\rho \left({\left\|r_{f}\left(z_{f_{j}}^{c_{i}},\chi \right)\right\|}_{\Sigma_{f_{j}}^{c_{i}}}^{2}\right)+\sum_{j\in L}\rho \left({\left\|r_{l}\left(z_{l_{j}}^{c_{i}},\chi \right)\right\|}_{\Sigma_{l_{j}}^{c_{i}}}^{2}\right)+\sum_{j\in P}\rho \left({\left\|r_{p}\left(z_{p_{j}}^{c_{i}},\chi \right)\right\|}_{\Sigma_{p_{j}}^{c_{i}}}^{2}\right)+\\ \sum_{k\in L,j\in P}\rho \left({\left\|r_{lp}\left(z_{l_{k},p_{j}}^{c_{i}},\chi \right)\right\|}_{\Sigma_{l_{k}}^{c_{i}},\Sigma_{p_{j}}^{c_{i}}}^{2}\right)+\sum_{k\in F,j\in P}\rho \left({\left\|r_{fp}\left(z_{f_{k},p_{j}}^{c_{i}},\chi \right)\right\|}_{\Sigma_{f_{k}}^{c_{i}},\Sigma_{p_{j}}^{c_{i}}}^{2}\right)+\sum_{m\in V,k\in L}\rho \left({\left\|r_{V}\left(z_{l_{k},V_{m}}^{c_{i}},\chi \right)\right\|}_{\Sigma_{l_{k}}^{c_{i}}}^{2}\right) \end{array}$$ (18)

where χ is the camera state vector that needs to be estimated in the $c_{i}$ frame. $r_{f}\left(z_{f_{j}}^{c_{i}},\chi \right)$, $r_{l}\left(z_{l_{j}}^{c_{i}},\chi \right)$, and $r_{p}\left(z_{p_{j}}^{c_{i}},\chi \right)$ are the point feature re-projection residual, the line feature re-projection residual and the plane feature re-projection residual, respectively. *F*, *L* and *P* are the sets of point features, line features and plane features observed by camera frames, respectively. $r_{fp}\left(z_{f_{k},p_{j}}^{c_{i}},\chi \right)$ and $r_{lp}\left(z_{l_{k},p_{j}}^{c_{i}},\chi \right)$ are the reprojection errors of point-to-plane distance and line-to-plane distance, respectively; $r_{V}\left(z_{l_{k},V_{m}}^{c_{i}},\chi \right)$ is the reprojection error based on the vanishing point constraint. The error equations for homogeneous features (point-point, line-line, and plane-plane) follow formulations presented in [34] and [43], and are not elaborated here due to space constraints. The remainder of this section focuses on the construction of vanishing point-based constraints and heterogeneous feature association residuals.

**Fig 14:**
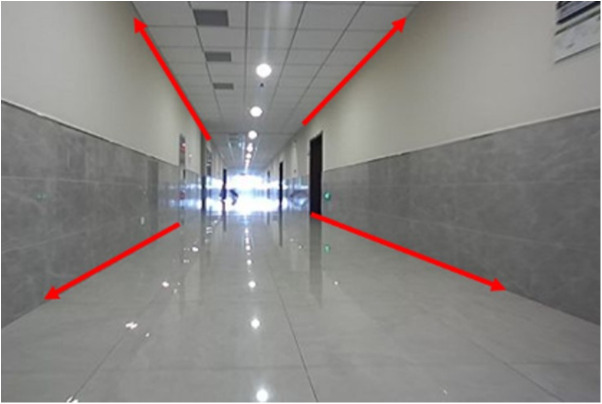
Scene of long corridor.

Previous works commonly used geometric or deep learning-based methods to extract vanishing points. However, both approaches are computationally intensive and often inaccurate in complex environments. For example, in narrow corridor scenes, only a single vanishing point is typically extractable, and extraction in other directions tends to be unstable, as illustrated in Fig 14.

**Fig 15:**
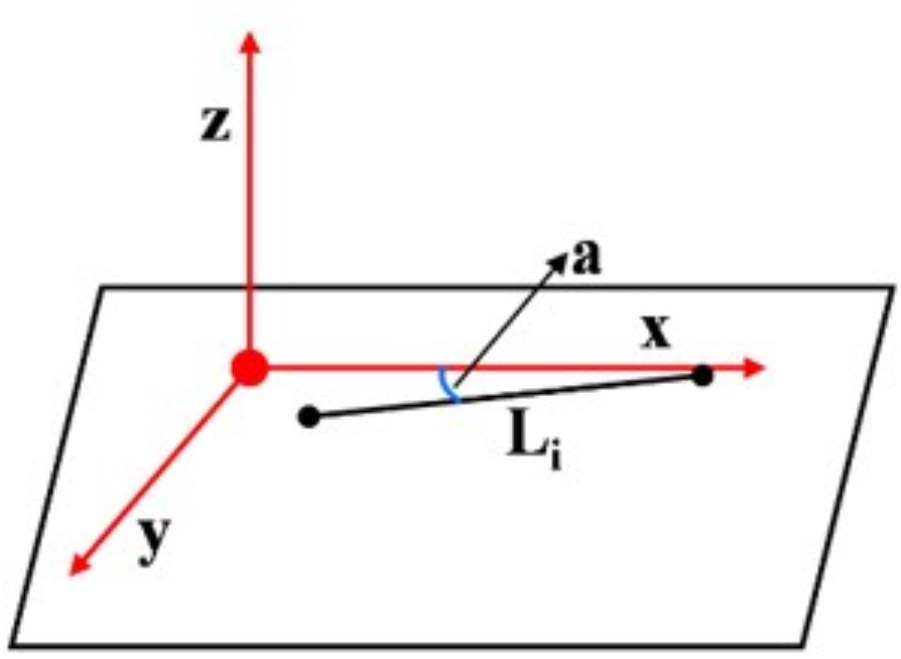
Line feature classification based on vanishing points.

To address this, we propose a vanishing point extraction method based on the normal vectors of orthogonal plane features. It is assumed that two or three orthogonal planes have already been detected. Let $V_{\text{1}}$, $V_{\text{2}}$ and $V_{\text{3}}$ denote the three mutually orthogonal prior vanishing point directions, corresponding to the X, Y, and Z axes of the Manhattan coordinate system. Observed line features are classified according to their directional alignment with these vanishing points. As shown in Fig 15, let a be the angle between a line feature $L_{i}$ and the X-axis. When a is less than 10 degrees, the line feature $L_{i}$ is considered to be aligned with the X-axis direction.

To simplify the computation, we first calculate the vector cross product between the direction vector of each line feature and the direction of a vanishing point. Line features that are approximately parallel to $V_{1}$、$V_{2}$ and $V_{3}$ are then assigned to corresponding directional categories by Eq. (19):


$$\delta =L_{k}⊗V_{m}$$ (19)

where $L_{k}$ is the direction vector of the kth line feature, and $V_{m}$ denotes the direction of the mth vanishing point. Ideally, δ should equal zero when the vectors are exactly parallel. However, due to noise and error propagation, δ is typically non-zero in practice. Therefore, a threshold is introduced: when δ≤0.05, the line feature $L_{k}$ is considered to belong to the vanishing point category $V_{m}$.

**Fig 16:**
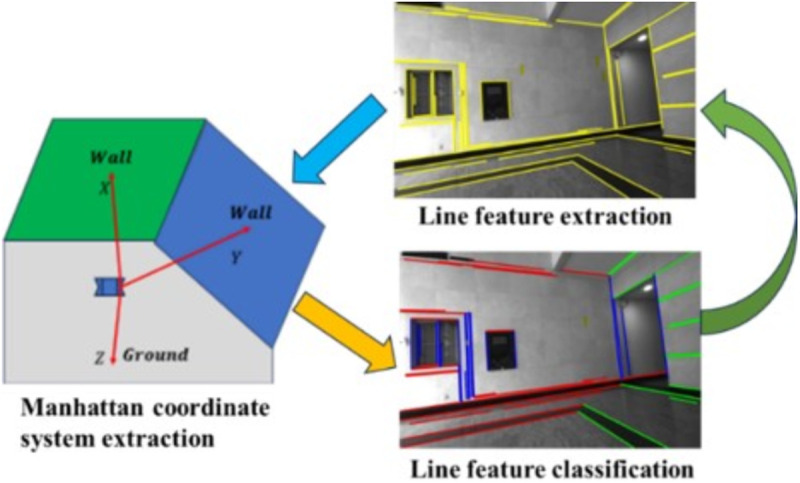
Iteration between Manhattan coordinate system and vanishing point.

When multiple orthogonal plane features are detected in the scene, a statistical analysis is performed to count how many line features are assigned to each candidate vanishing point. The vanishing point direction with the largest number of associated line features is retained as the final dominant vanishing point direction. The complete procedure is illustrated in Fig 16.

Following the method in [22], the structure-constrained residual equation based on vanishing points is defined as:


$$r_{V}\left(z_{l_{k},V_{m}}^{c_{i}},\chi \right)={\sum {\mathrm{\backslash nolimits}}_{k=0}^{L}\left\|L_{k}^{c_{i}}⊗V_{m}\right\|}^{2}$$ (20)

Since $r_{V}\left(z_{l_{k},V_{m}}^{c_{i}},\chi \right)$ is solely dependent on the rotation matrix $R_{c_{i}}^{w}$, this matrix can be estimated through nonlinear optimization using Eq. (20). The translation matrix $t_{c_{i}}^{w}$ is subsequently obtained through additional geometric constraints. This approach effectively decouples the rotation and translation matrices, facilitating faster convergence of the optimization process and reducing the risk of convergence to local minima. To ensure consistency with point feature residual optimization, we adopt a two-endpoint representation for line features. As referenced in [16], a line feature can be represented using its two endpoints in the world coordinate system, as show in Eq. (21):


$$L_{k}^{c_{i}}={\left(R_{c_{i}}^{w}\right)}^{T}\left(p_{k,e}^{w}-p_{k,s}^{w}\right)$$ (21)

The error equation based on the vanishing point constraint can be rewritten as:


$$\gamma_{V}\left(p_{k,e}^{w},p_{k,s}^{w},T_{c_{i}}^{W}\right)=L_{k}^{c_{i}}⊗V_{m}$$ (22)

The Jacobian matrix of $\gamma_{V}$ with respect to the state vector χ can be written as:


$$\frac{\partial \gamma_{V}}{\partial \chi }=\frac{\partial \gamma_{V}}{\partial L_{k}^{c_{i}}}\left(\frac{\partial L_{k}^{c_{i}}}{\partial \delta \xi }+{\left(R_{c_{i}}^{w}\right)}^{T}\left(\frac{\partial \left(p_{k,e}^{w}-p_{k,s}^{w}\right)}{\partial \delta p_{k,e}^{w}}+\frac{\partial \left(p_{k,e}^{w}-p_{k,s}^{w}\right)}{\partial \delta p_{k,s}^{w}}\right)\right)+\frac{\partial \gamma_{V}}{\partial \delta V_{m}}=-{\left[V_{m}\right]}^{\times }\left(\frac{\partial L_{k}^{c_{i}}}{\partial \delta \xi }+{\left(R_{c_{i}}^{w}\right)}^{T}\left(\frac{\partial p_{k,e}^{w}}{\partial \delta p_{k,e}^{w}}-\frac{\partial p_{k,s}^{w}}{\partial \delta p_{k,s}^{w}}\right)\right)$$ (23)

where $V_{m}$ is a constant, $\frac{\partial \gamma_{V}}{\partial \delta V_{m}}$=0. The perturbation model can be used to solve $\frac{\partial L_{k}^{c_{i}}}{\partial \delta \xi }$:


$$\frac{\partial L_{k}^{c_{i}}}{\partial \delta \xi }={\left[R_{c_{i}}^{w}\left(p_{k,e}^{w}-p_{k,s}^{w}\right)\right]}^{\times }$$ (24)

where $p_{k,e}^{w}$ and $p_{k,s}^{w}$ are the endpoints of the line $L_{k}$ in the world coordinate system. Let ξ represent the Lie algebra corresponding to the transformation from the camera frame $c_{i}$ to the world frame. The matrices $R_{c_{i}}^{w}$ and $t_{c_{i}}^{w}$ denote the rotation and translation from the camera coordinate system to the world coordinate system, respectively. The symbol $[{]}^{\times }$ denotes the skew-symmetric matrix operator.

## 4 Experimental verification and evaluation

This study introduces PLPM-SLAM, a novel tightly coupled RGB-D SLAM system that integrates point-line-plane collaborative optimization with Manhattan plane feature constraints. To address scenarios where only two orthogonal Manhattan plane features are detected—rendering translation matrix decoupling infeasible—we propose a translation matrix decoupling algorithm assisted by hypothetical planes. During feature tracking, both homogeneous (point-point, line-line, plane-plane) and heterogeneous (point-line, point-plane, line-plane) feature associations are considered. Plane feature matching is performed using a descriptor constructed from the distances between nnn (where n > 3n > 3n > 3) non-collinear shared keypoints and planar features in both the current and reference frames. In unstructured environments, we jointly optimize homogeneous feature matching errors and heterogeneous feature association errors, and propose a SLAM model that integrates vanishing point constraints with point-line-plane collaborative optimization.

To evaluate the effectiveness of the proposed system, we conducted experiments using both public datasets—TUM RGB-D and ICL-NUIM—and real-world data collected with a ZED2i RGB-D camera. The TUM RGB-D dataset is a widely used benchmark for evaluating visual SLAM and visual odometry (VO) systems. It provides synchronized RGB and depth images at a resolution of 640 × 480 and a frame rate of 30 Hz, along with high-precision ground truth camera trajectories. We selected sequences with prominent planar structures to assess our system’s performance. The ICL-NUIM dataset offers synthetic indoor environments, such as living rooms and office spaces, which are ideal for evaluating point-plane SLAM frameworks. It also includes ground truth trajectories, enabling detailed quantitative analysis.

All experiments were conducted on a laptop equipped with an Intel i7 CPU, 8 GB RAM, and no dedicated GPU. We compared our proposed SLAM system against several state-of-the-art RGB-D SLAM frameworks: ORB-SLAM3 is a classical point-feature-based SLAM system that supports monocular, stereo, and RGB-D inputs. Its stable performance and high accuracy have made it a standard baseline in SLAM research; MSC-VO is a visual SLAM system that integrates point and line features, as well as structural constraints. The use of line features improves robustness in low-texture environments, and Manhattan coordinate constraints help reduce long-term error accumulation; Planar-SLAM enhances tracking and mapping accuracy by leveraging geometric primitives such as points, lines, and planes. It employs a decoupled optimization strategy and introduces an additional pose optimization module based on Manhattan relationships. However, its performance degrades in scenes where reliable Manhattan structures cannot be detected; Manhattan-SLAM estimates orthogonal planes to recover Manhattan frames and supports real-time pose estimation in both Manhattan and non-Manhattan environments.

A module-level comparison of these representative systems is provided in Table 1. In the Table 1, “Heterogeneous feature distance” refers to constraints involving cross-feature associations (e.g., point-to-plane, line-to-plane), while “Homogeneous structural constraints” describe spatial relationships among same-type features (e.g., line-line, plane-plane). To evaluate SLAM performance, we use the Root Mean Square Error (RMSE) of the Absolute Trajectory Error (ATE) as the primary accuracy metric.

**Table 1:** The module comparison of related classical algorithms.

Function\Model	ORB-SLAM3 [15]	MSC-VO [16]	Planar-SLAM [17]	Manhattan-SLAM [18]	UPLP-SLAM [35]	PLPM-SLAM
Point	**√**	**√**	**√**	**√**	**√**	**√**
Line		**√**	**√**	**√**	**√**	**√**
Plane			**√**	**√**	**√**	**√**
Heterogeneous feature distance constraints					**√**	**√**
Homogeneous feature structure constraints		**√**	**√**	**√**	**√**	**√**
Able to handle both unstructured and structured scenarios	**√**	**√**		**√**	**√**	**√**
Decoupled Rotation Matrix			**√**	**√**		**√**
Decoupled Translation Matrix						**√**

### 4.1 A public dataset validation (TUM and ICL-NUIM)

**Fig 17:**
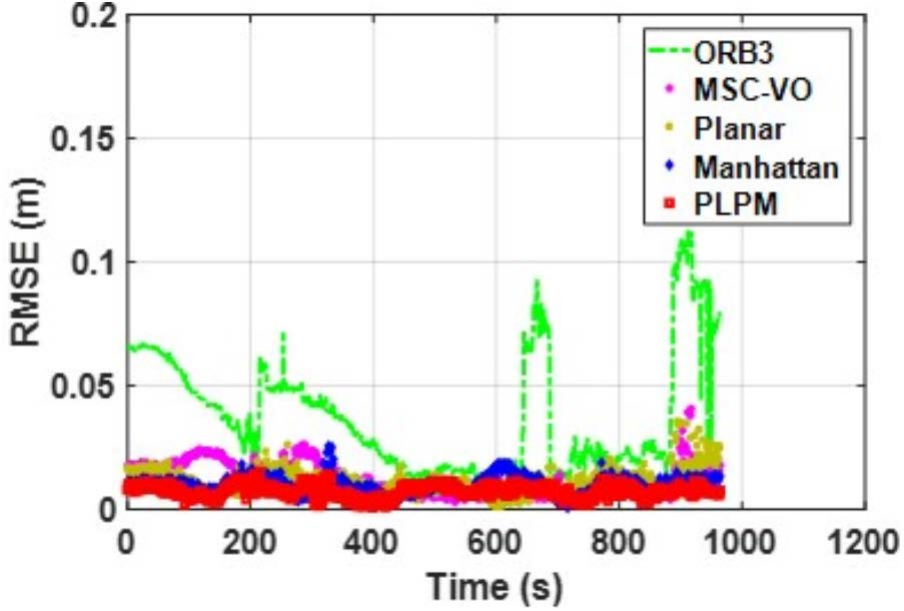
RMSE comparison of different algorithms in the ICL dataset living_room_traj1_frei.

**Fig 18:**
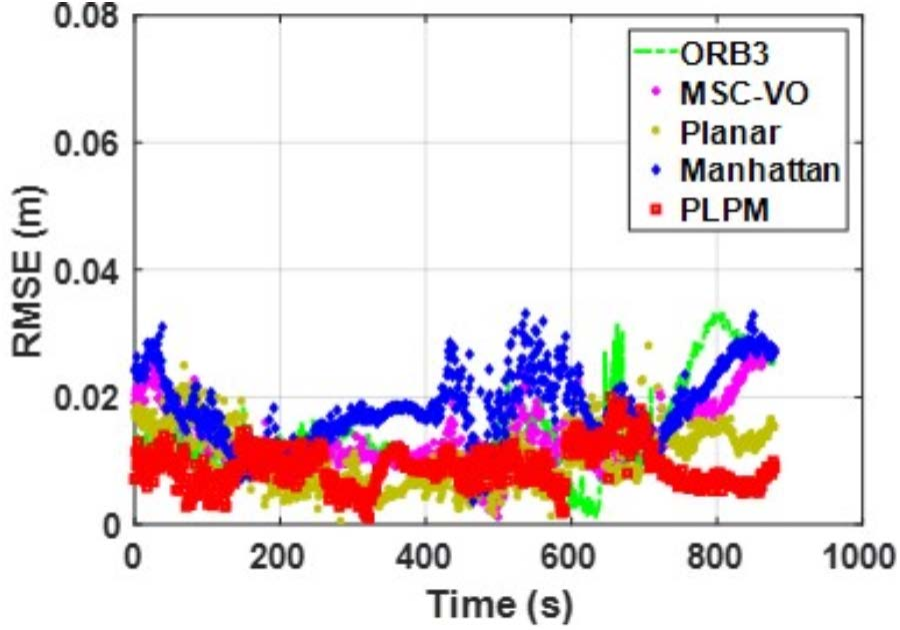
RMSE comparison of different algorithms in the ICL dataset living_room_traj2_frei.

**Fig 19:**
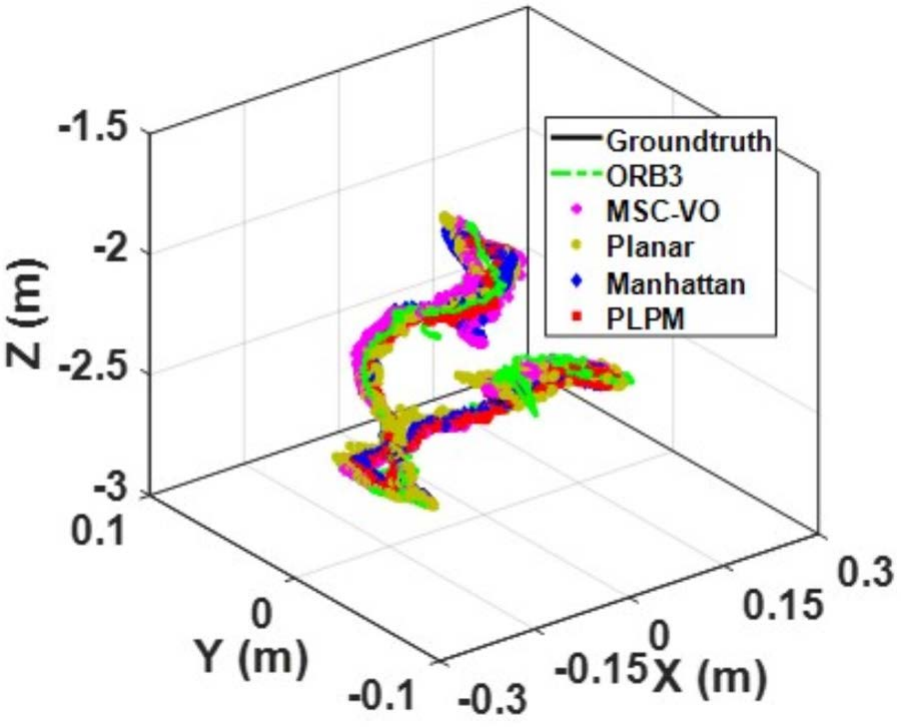
Trajectory comparison of different algorithms in the ICL dataset living_room_traj1_frei.

**Fig 20:**
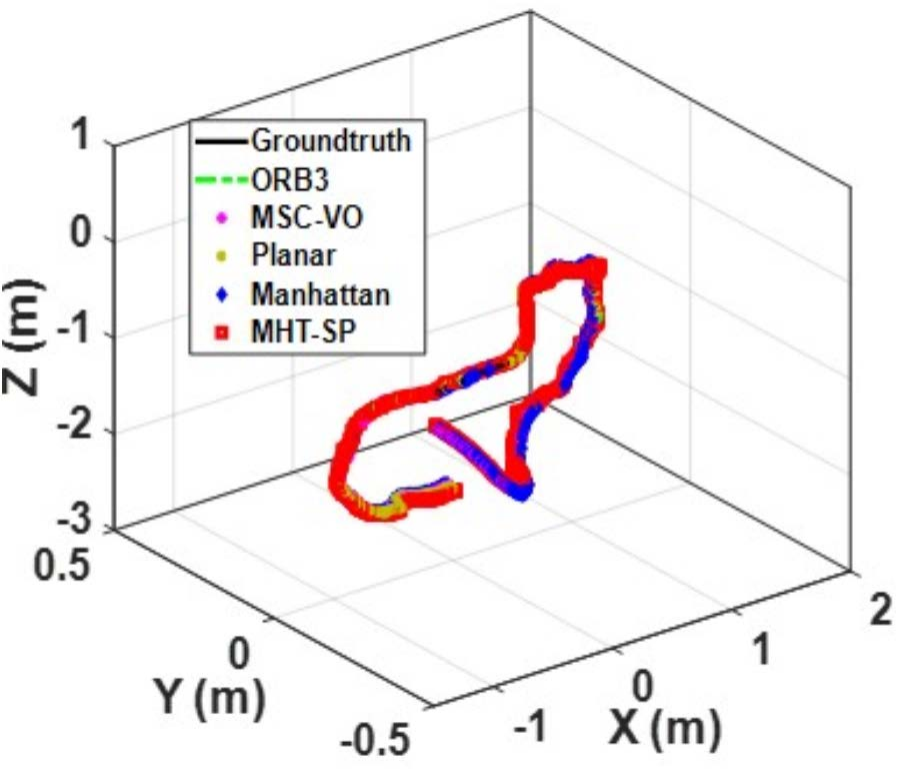
Trajectory comparison of different algorithms in the ICL dataset living_room_traj2_frei.

We first tested using the public datasets ICL-NUIM and TUM. For the ICL-NUIM dataset, the paper only used the bedroom scene. Table 2 compares the results of several RGB-D SLAM methods on these two datasets. PLPM-SLAM is the algorithm proposed in this paper. Compared with PLPM-SLAM, PLP-SLAM removes the vanishing point constraint in the backend optimization. The RMSE results for several ICL-NUIM datasets are presented in Figs 17 and 18, while the trajectory comparisons for these datasets are illustrated in Figs 19 and 20.

**Table 2:** Evaluation results of translation ATE RMSE (unit: m) on ICL-NUIM and TUM datasets. Bold numbers represent the best performances. ‘*×*’ represents tracking lost.

Type	Dataset name	ORB-SLAM3 [15]	Manhattan-SLAM [18]	Planar-SLAM [17]	MSC-VO [16]	Ours		
						PLP-SLAM	PLPM-SLAM	Increase (%)
TUM	1_desk	0.0224	0.0271	*×*	0.0220	0.0167	**0.0139**	37.95
	1_floor	0.0194	0.0351	0.0962	0.0449	0.0135	**0.0114**	41.24
	1_rpy	0.0204	0.0176	*×*	0.0244	0.0158	**0.0132**	35.29
	1_xyz	0.0114	0.0118	0.0172	0.0131	0.0144	**0.0103**	9.65
	2_desk_with_person	0.0229	0.0107	*×*	0.0214	0.0154	**0.0091**	60.26
	2_pioneer_slam3	0.0290	0.0853	0.8662	*×*	0.0239	**0.0216**	25.52
	2_xyz	0.0101	0.0100	0.0086	0.0166	0.0079	**0.0042**	58.42
	3_long_office_household	0.0139	0.0223	0.0363	0.0172	0.0163	**0.0135**	2.88
	3_structure_texture_far	0.0104	0.0138	0.0142	0.0143	0.0098	**0.0087**	16.35
	3_structure_texture_near	0.0112	0.0120	0.0202	0.0162	0.0107	**0.0097**	13.39
ICL	living_room_traj1_frei	0.0447	0.0102	0.0123	0.0141	0.0102	**0.0077**	82.77
	living_room_traj2_frei	0.0160	0.0191	0.0115	0.0146	0.0113	**0.0094**	41.25
	living_room_traj3_frei	0.0109	0.0154	**0.0061**	0.0397	0.0099	0.0073	33.03

In the ICL-NUIM dataset, the living room sequences feature relatively small scenes with clear and regular structural characteristics. The main directions of the MF remain consistently stable. Since this scene strictly adheres to the MF, Planar-SLAM demonstrates superior performance compared to other algorithms. Notably, in the living_room_traj3_frei sequence, Planar-SLAM achieves the best results among all tested algorithms. In the living_room_traj1_frei and living_room_traj2_frei sequences, Planar-SLAM also exhibits commendable performance.

**Fig 21:**
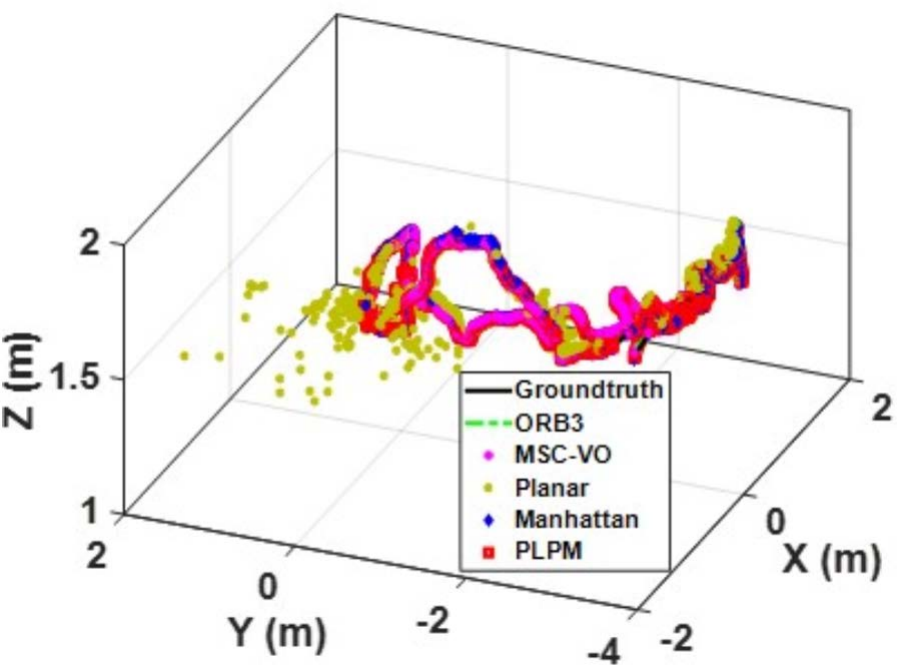
Trajectory comparison of different algorithms in the TUM dataset 2_desk_with_person.

**Fig 22:**
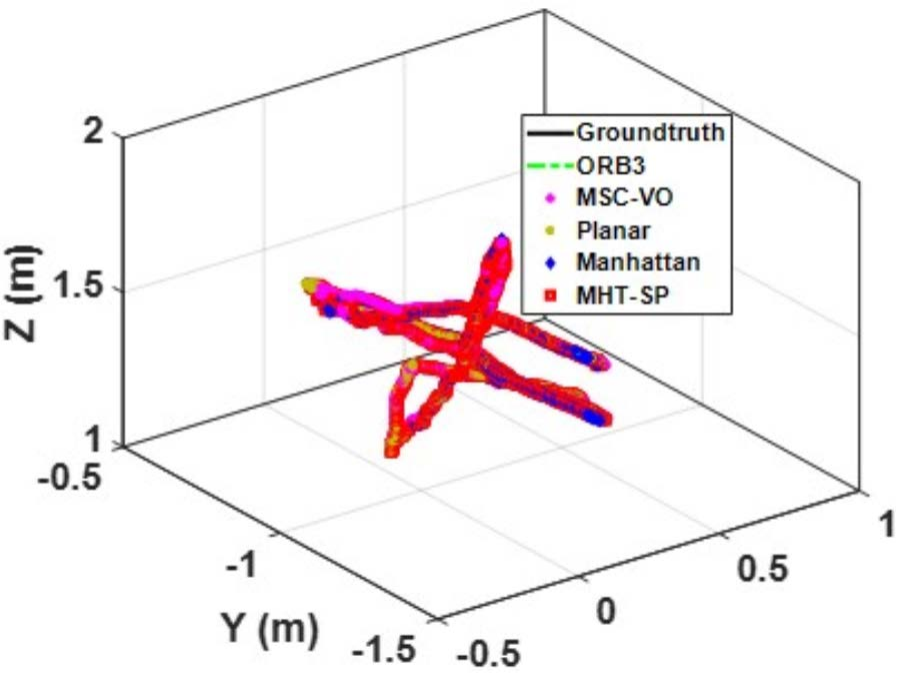
Trajectory comparison of different algorithms in the TUM dataset 2_xyz.

**Fig 23:**
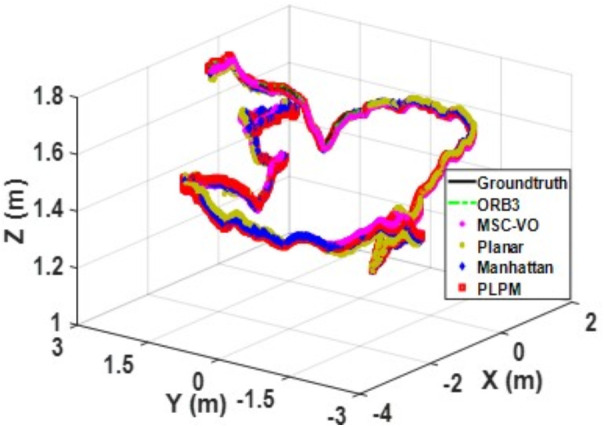
Trajectory comparison of different algorithms in the TUM dataset 3_long_office_household.

**Fig 24:**
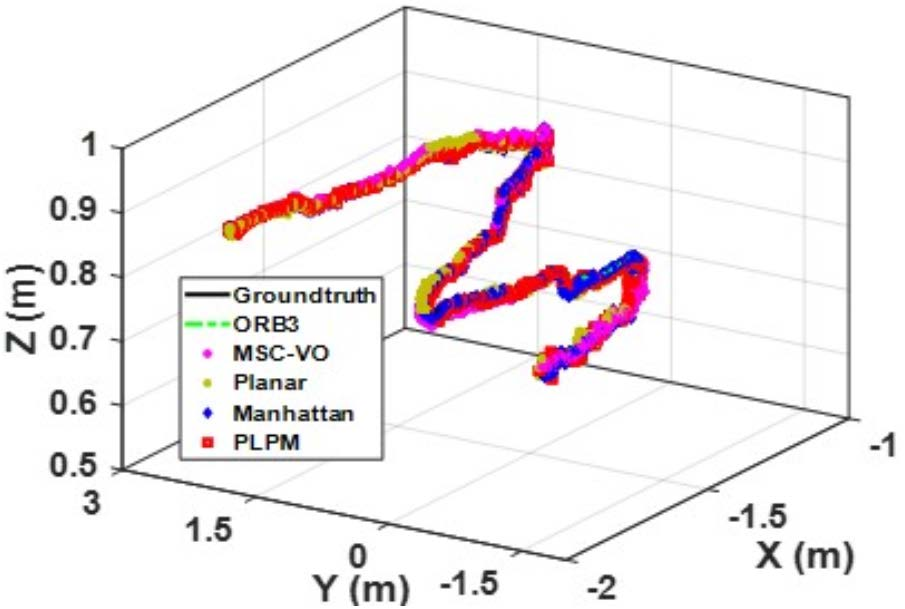
Trajectory comparison of different algorithms in the TUM dataset 3_structure_texture_near.

**Fig 25:**
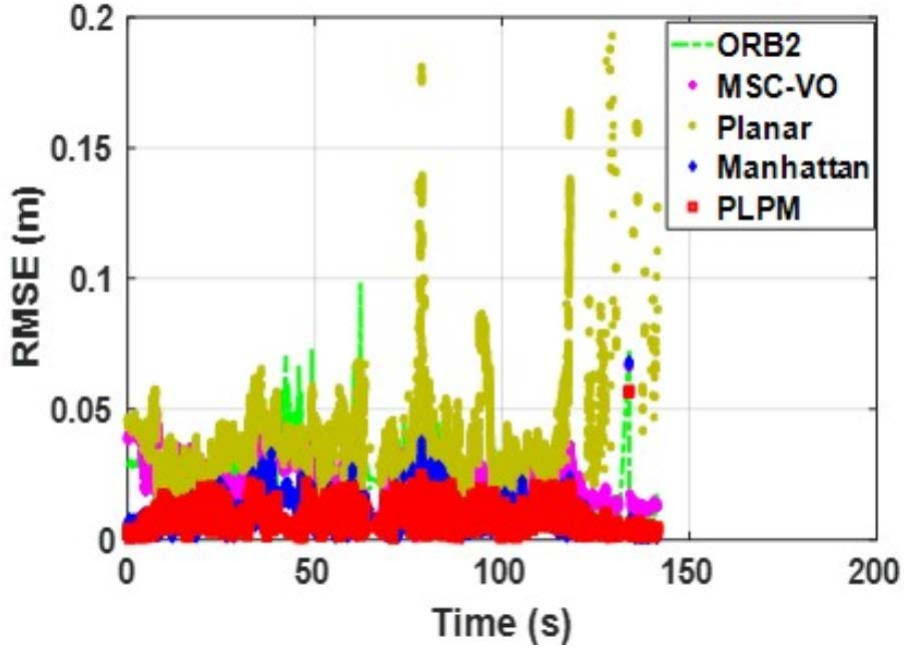
RMSE comparison of different algorithms in the TUM dataset 2_desk_with_person.

**Fig 26:**
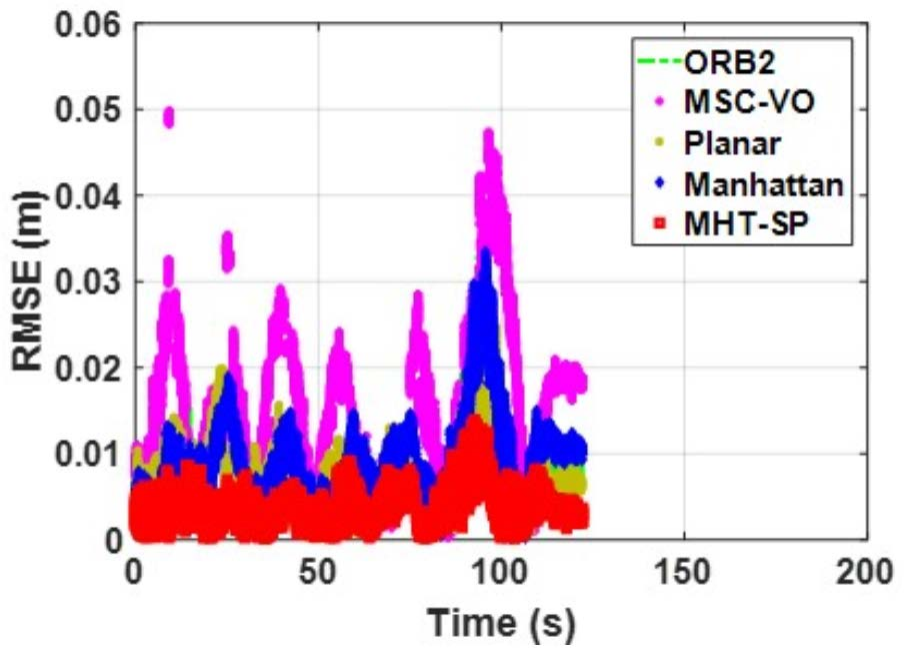
RMSE comparison of different algorithms s in the TUM dataset 2_xyz.

**Fig 27:**
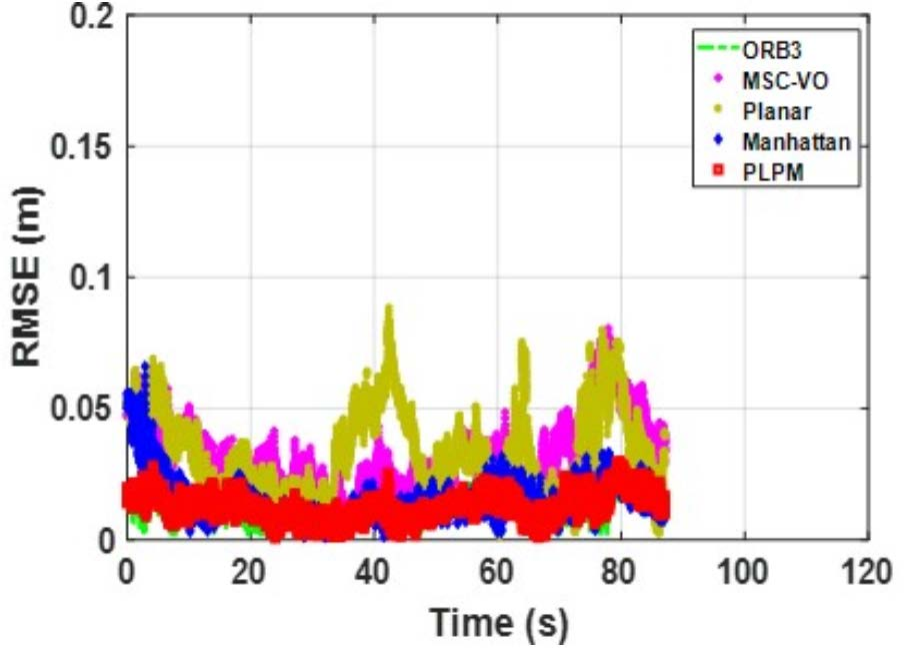
RMSE comparison of different algorithms in the TUM dataset 3_long_office_household.

**Fig 28:**
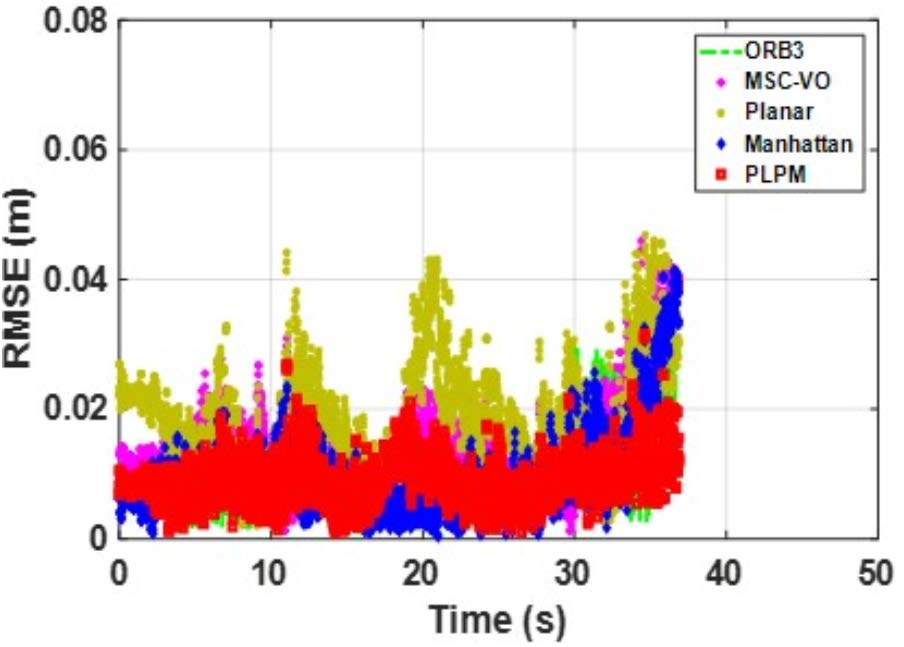
RMSE comparison of different algorithms in the TUM dataset 3_structure_texture_near.

As shown in the table, in the living_room_traj1_frei sequence, where the structural features of the scene are prominent but texture features are not, the RMSE of ORB-SLAM3, which relies on point features, is significantly higher than the RMSE of other algorithms based on structural constraints. Across these three sequences, the RMSE of Manhattan-SLAM is slightly higher than that of Planar-SLAM. PLPM-SLAM, however, demonstrates outstanding performance, achieving the best results in the living_room_traj1_frei and living_room_traj2_frei sequences and the second-best result in the living_room_traj3_frei sequence. Next, we analyze the TUM dataset. The trajectory results for several TUM datasets are presented in Figs 21–24, while the RMSE comparisons for these datasets are illustrated in Figs 25–28.

Based on the RMSE comparison across the TUM dataset, PLPM-SLAM consistently outperforms all other methods. Whether in structurally rich sequences such as 3_structure_texture_far and 3_structure_texture_near, or in low-texture scenarios like 1_xyz, PLPM-SLAM demonstrates stable and reliable performance, as shown in Table 2. Notably, in the 2_desk_with_person and 2_xyz sequences, PLPM-SLAM reduces RMSE by nearly 60% compared to ORB-SLAM3. For Planar-SLAM, the results indicate weaker performance across most sequences, with the exception of 2_xyz, where it performs relatively well. However, tracking failures are observed in the 1_desk and 1_rpy sequences. In contrast, Manhattan-SLAM shows robust performance in the majority of sequences, except for 2_pioneer_slam3, where its performance slightly declines. Importantly, Manhattan-SLAM does not exhibit tracking loss in any of the evaluated sequences.

The RMSE of MSC-VO is generally comparable to that of ORB-SLAM3, though slightly higher in most cases, except for 2_desk_with_person and 1_desk, where it achieves marginal improvements. As shown in Table 2, the proposed PLPM-SLAM achieves the best overall performance across the TUM dataset. While its advantage is less pronounced in sequences such as 2_pioneer_slam3 and 3_long_office_household, it demonstrates significant superiority in most other cases.

In sequences with strong structural and texture features, such as living_room_traj3_frei, structure_texture_far, and structure_texture_near, all methods perform well. In low-structure, high-texture scenes (e.g., 2_pioneer_slam3), PLPM-SLAM and ORB-SLAM3 excel. However, due to chaotic line features in such scenes, MSC-VO, which relies heavily on line features for structural constraints, suffers from tracking loss. In high-structure, low-texture environments, all methods except ORB-SLAM3 maintain strong performance—for instance, in living_room_traj1_frei. In dynamic environments, feature-point-based systems like ORB-SLAM3 are more vulnerable to dynamic object interference, while line- and plane-feature–based systems, including PLPM-SLAM, show greater robustness (e.g., in 2_desk_with_person).

Table 3 reports the number of frames in which Manhattan Frames (MF) were successfully extracted in the experimental datasets. “MF” refers to the classical approach relying on the detection of three mutually orthogonal planes. “SMF” denotes the method proposed in this study, where the third orthogonal plane is constructed with the assistance of shared feature points between the current and previous frames when only two orthogonal planes are initially detected.

**Table 3:** Frame counts for MF extraction in the experimental datasets.

Type	Dataset name	Is the structure obvious?	Frame count		
			MF	SMF	Total frame
TUM	1_desk	Not obvious	21	63	829
	1_floor	Not obvious	38	221	1437
	1_rpy	Not obvious	12	108	977
	1_xyz	Not obvious	3	122	899
	2_desk_with_person	Not obvious	67	1882	4854
	2_pioneer_slam3	Not obvious	108	212	1867
	2_xyz	Not obvious	121	1706	4374
	3_long_office_household	Not obvious	54	692	2507
	3_structure_texture_far	Obvious	604	810	906
	3_structure_texture_near	Obvious	572	803	1044
ICL	living_room_traj1_frei	Obvious	681	865	966
	living_room_traj2_frei	Obvious	770	832	881
	living_room_traj3_frei	Obvious	1041	1134	1241

**Fig 29:**
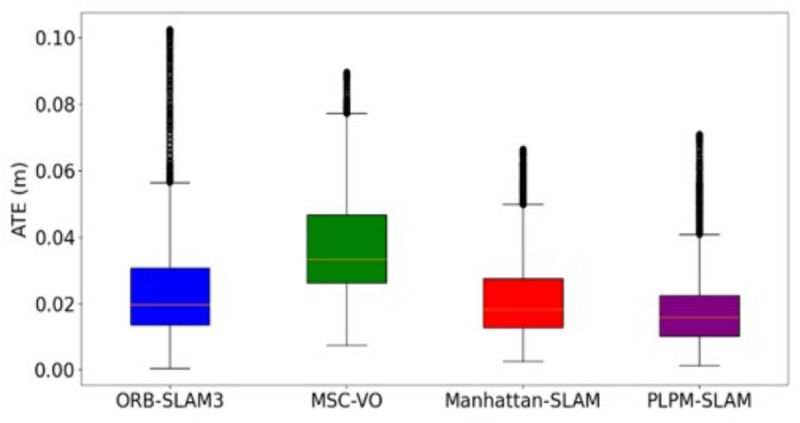
Box plot of ATE of 1_rpy for different algorithms.

**Fig 30:**
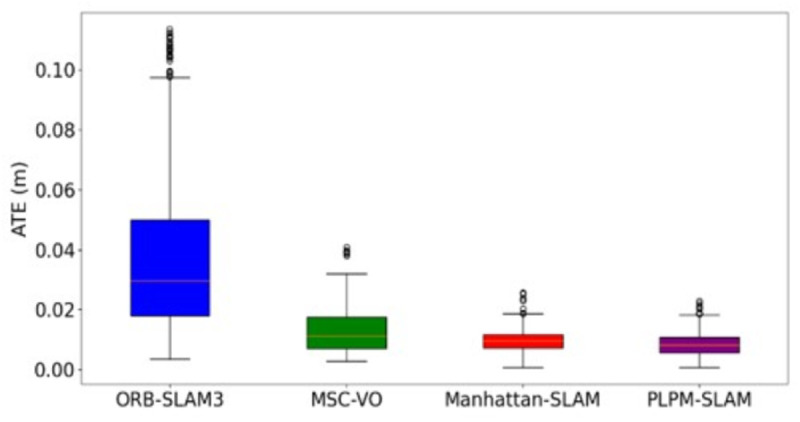
Box plot of ATE of living_room_traj1_frei for different algorithms.

Given that a greater number of MFs introduces more geometric constraints, the effectiveness of backend optimization can be significantly enhanced. As shown in Table 3, when the scene exhibits strong structural features, both the classical method and the proposed method generate a comparable number of MFs. However, in environments with less obvious structural characteristics, the proposed method detects significantly more MFs than the classical approach. For instance, in the 1_xyz sequence, the desktop computer and the desk are nearly perpendicular. The classical method identifies only two mutually perpendicular MPs, which is insufficient to construct a complete MF. Consequently, it is able to decouple only the rotation matrix, while the translation matrix remains entangled. In contrast, the proposed method leverages shared keypoints across frames to construct a virtual auxiliary plane, enabling the extraction of three orthogonal MPs. This allows for complete MF construction and the decoupling of both the rotation and translation matrices. A similar improvement is observed in the 2_desk_with_person sequence. Figs 29 and 30 present box plots of the Absolute Trajectory Error (ATE) for different algorithms on the 1_rpy and living_room_traj1_frei datasets, further illustrating the performance advantage of the proposed method. The 1_rpy sequence contains weak structural cues, whereas living_room_traj1_frei exhibits more distinct structural features. As shown in Figs 29 and 30, PLPM-SLAM consistently achieves the lowest median ATE and the smallest variance across both scenarios, demonstrating superior accuracy and robustness. Manhattan-SLAM ranks second, with a moderate error spread and fewer outliers. In contrast, ORB-SLAM3 yields the poorest performance, characterized by large error fluctuations and numerous outliers, indicating reduced stability under varying conditions.

**Fig 31:**
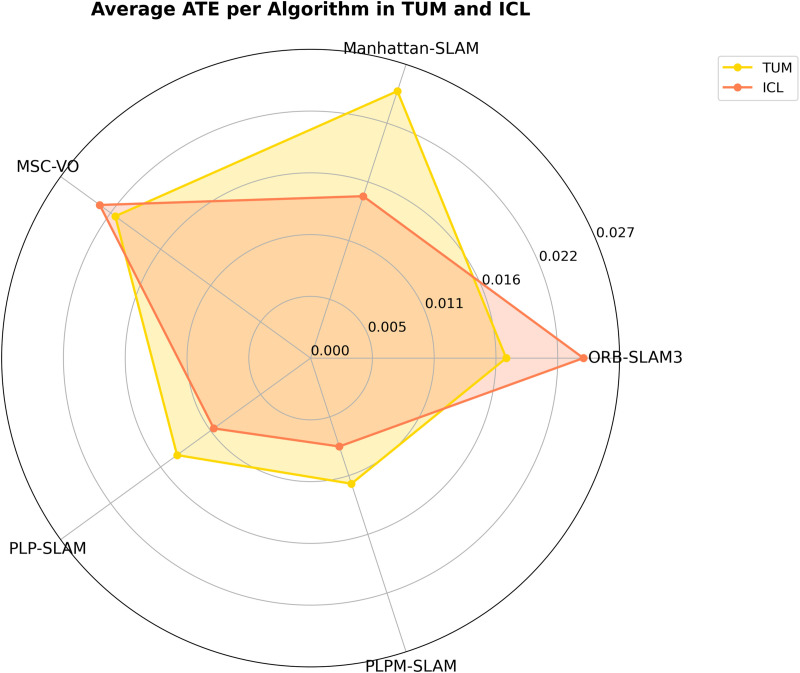
Average ATE Comparison of SLAM Algorithms on TUM and ICL Datasets.

Fig 31 presents a radar chart comparing the average ATE of each SLAM algorithm across the TUM and ICL datasets. The chart highlights the overall performance of six visual SLAM systems, where smaller values represent better accuracy. On the TUM dataset, PLPM-SLAM significantly outperforms all other methods, followed by PLP-SLAM, demonstrating strong adaptability to dynamic and low-structure environments. Similarly, on the ICL dataset, PLPM-SLAM achieves the lowest average ATE, confirming its robust and consistent performance across a wide range of sequences.

**Fig 32:**
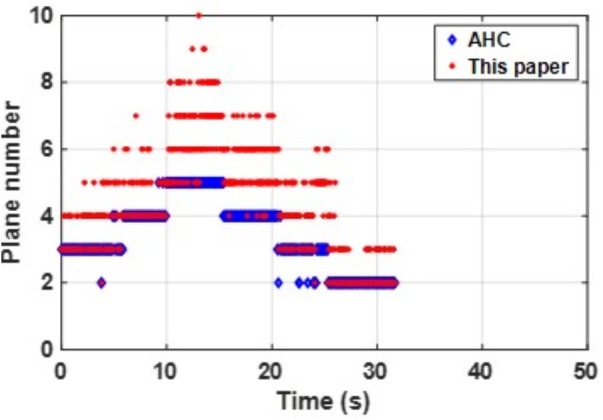
Comparison of the number of plane feature extractions between scenarios in structure_texture_far.

**Fig 33:**
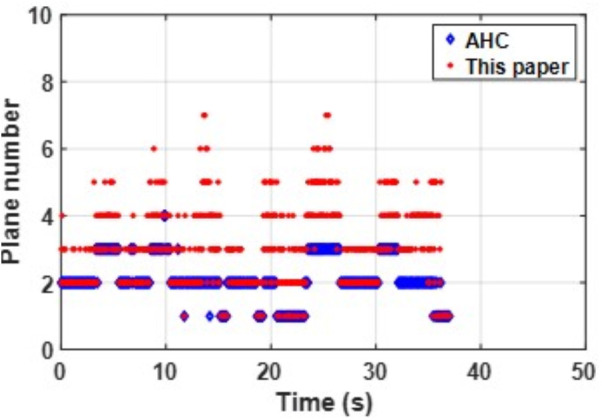
Comparison of the number of plane feature extractions between scenarios in structure_texture_near.

The consistent best performance of PLPM-SLAM in both datasets indicates its strong generalization capability and trajectory accuracy in both structured and unstructured indoor scenes. As shown in the Figs 32 and 33 below, compared to the AHC algorithm, the proposed method can extract richer plane features, which provide more constraints during backend nonlinear optimization and offer more MP features.

**Fig 34:**
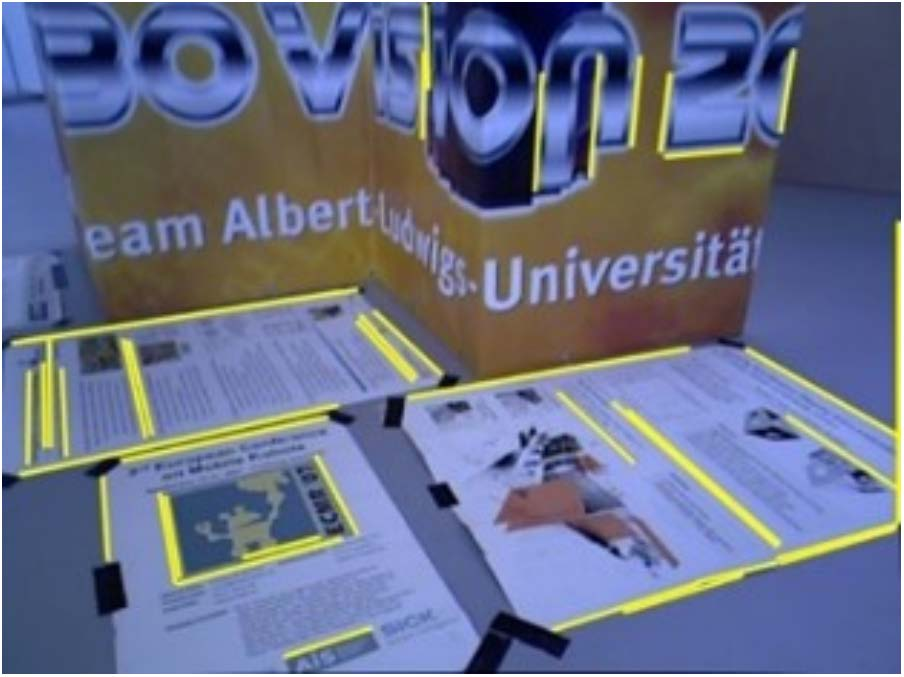
Extraction of line features in the structure_texture_far sequence.

**Fig 35:**
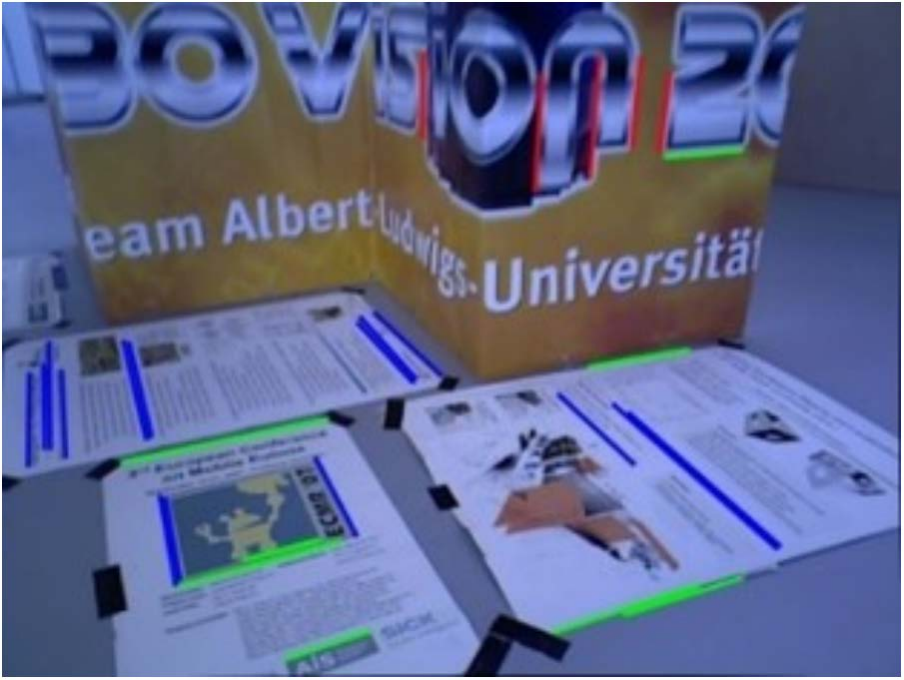
Classification based on vanishing points in the structure_texture_far sequence.

**Fig 36:**
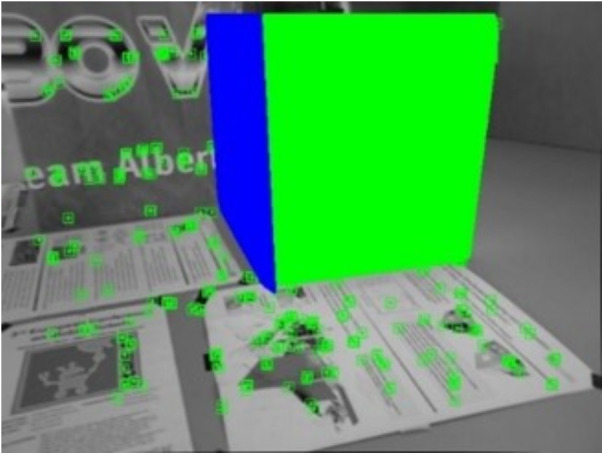
Orthogonal plane features extracted from the current frame in the structure_texture_far sequence.

**Fig 37:**
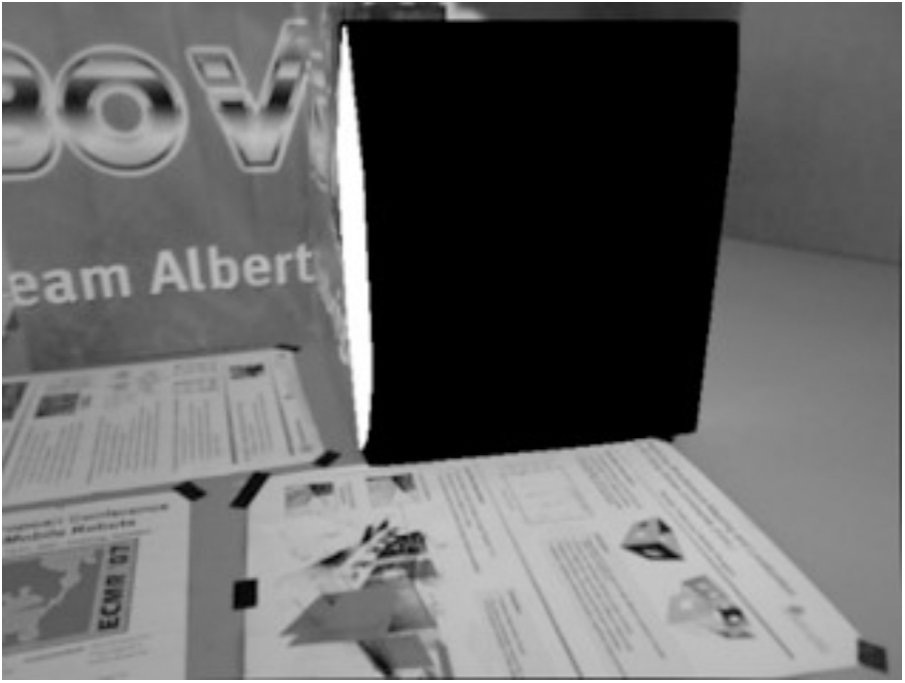
Orthogonal plane features extracted from orthogonal plane features matched in the historical frame in the structure_texture_far sequence.

Figs 34 and 35 present the extracted line features and their classification based on vanishing point estimation. As shown in Fig 35, the proposed method for vanishing point estimation using orthogonal plane features proves to be effective and reliable. Figs 36 and 37 illustrate the orthogonal plane features extracted from the current frame, along with those matched from the historical frames. The image index of the current frame is 230, and the matched historical frame is indexed at 101. This indicates that the pose estimation of the current frame depends solely on the pose accuracy of frame 101, rather than the accumulated accuracy of all intermediate frames. As a result, pose estimation errors between the current and historical frames do not propagate or accumulate, enhancing the system’s robustness to drift over time.

### 4.2 Verification of Actual Test Scenes Based on Zed2i Camera

**Fig 38:**
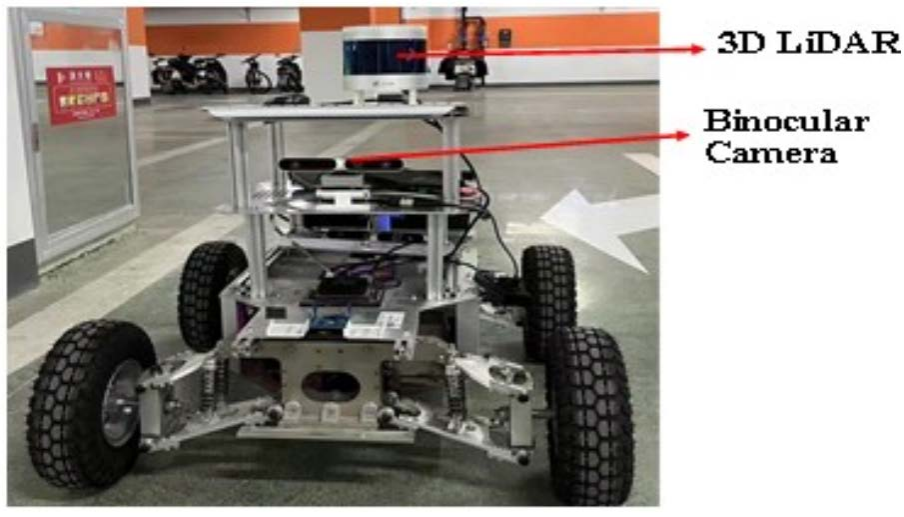
ZED2i camera-based test equipment.

**Fig 39:**
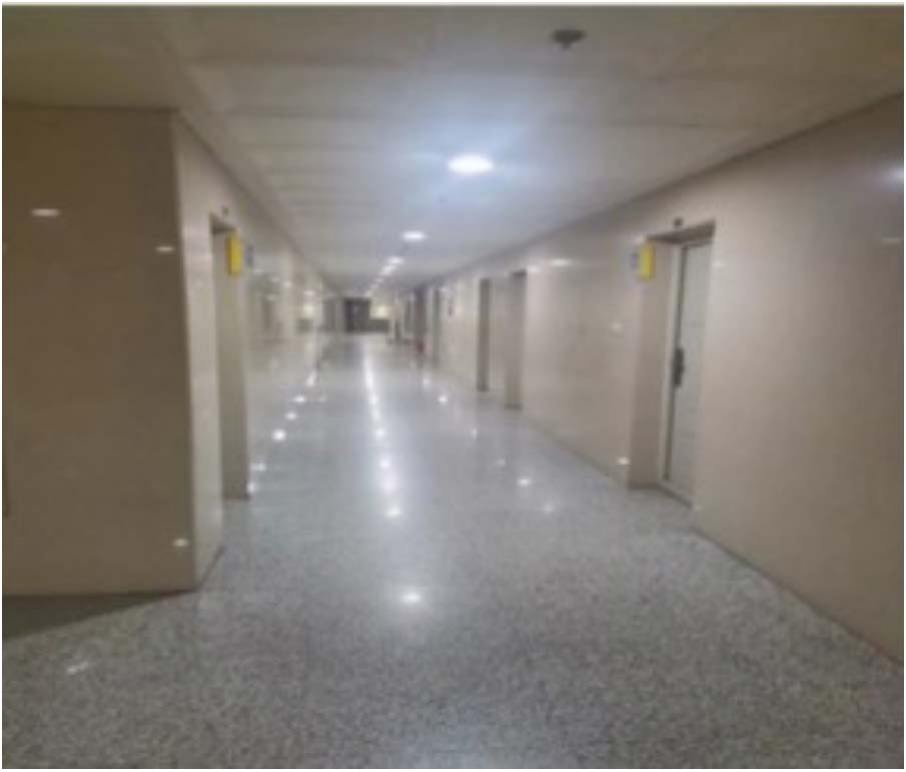
Scene of long corridor scene.

**Fig 40:**
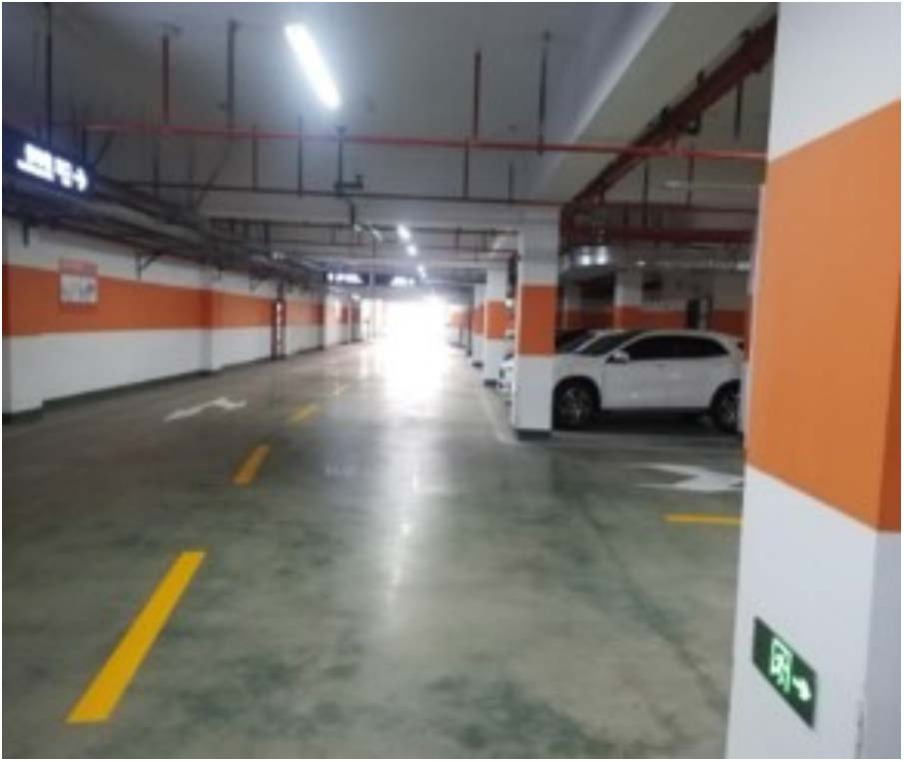
Scene of underground parking scene.

To further evaluate the effectiveness of the proposed model—based on Manhattan plane feature constraints and point–plane collaborative optimization—we constructed a custom experimental platform equipped with a ZED2i RGB-D camera and a 3D LiDAR sensor. The extrinsic parameters between the two sensors were carefully calibrated, as illustrated in Fig 38. The 3D LiDAR localization results were used as ground truth for evaluation. Using this setup, we conducted experiments in two real-world environments at Changzhou University: the third-floor corridor and the basement-level parking garage of the Ligong Building, as illustrated in Figs 39 and 40.

1. Dataset s 1-1, 1-2, and 1-3 were collected in the long corridor on the third floor of Ligong Building at Changzhou University.
2. Dataset s 2-1, 2-2, and 2-3 were collected in the basement level one parking garage of Ligong Building at Changzhou University.

**Fig 41:**
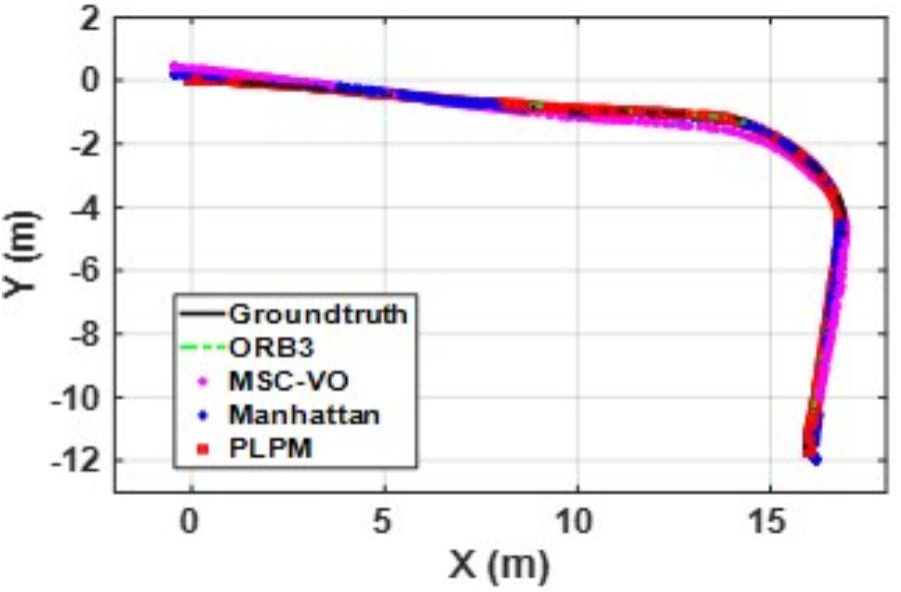
Trajectory comparison of Data_1-1 based on the actual scene measured by ZED2i camera.

**Fig 42:**
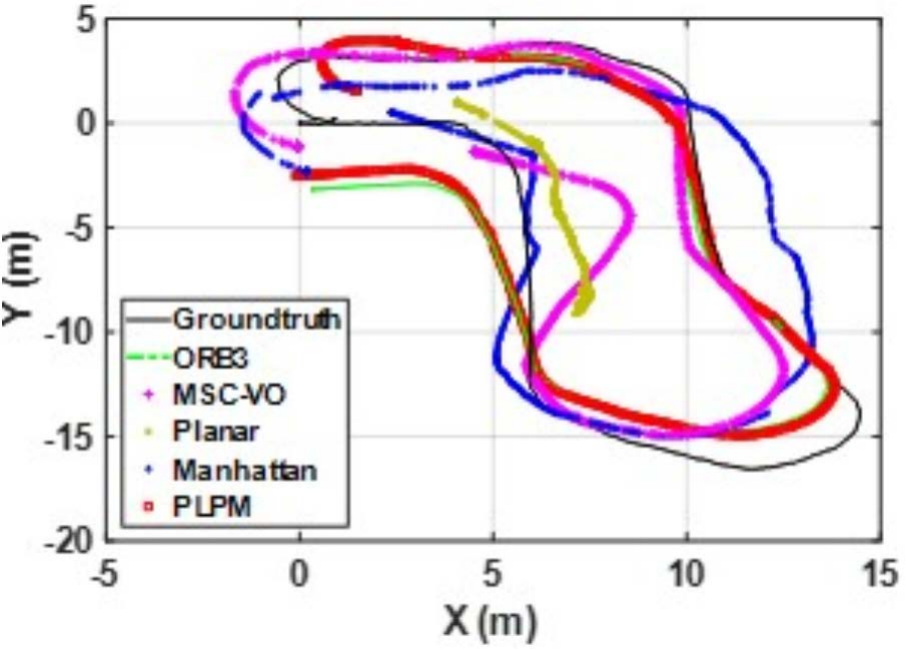
Trajectory comparison of Data_2-2 based on the actual scene measured by ZED2i camera.

**Fig 43:**
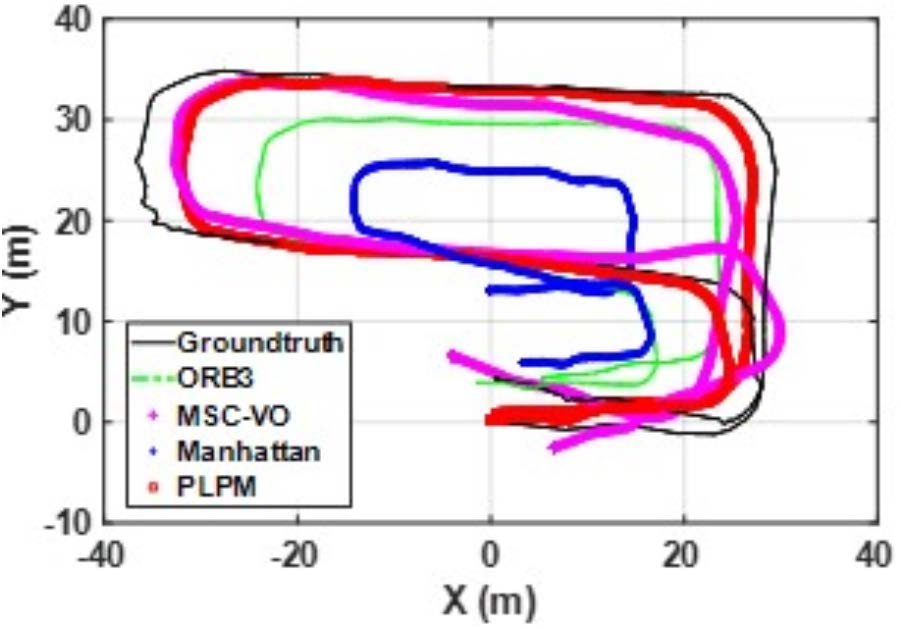
Trajectory comparison of Data_2-3 based on the actual scene measured by ZED2i camera.

**Fig 44:**
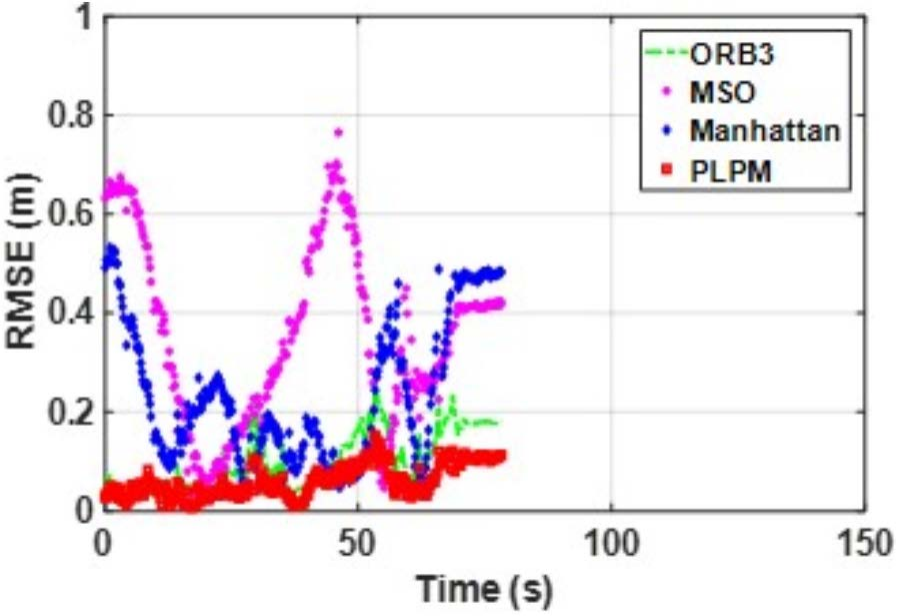
RMSE comparison of Data_1-1 based on the actual scene measured by ZED2i camera.

**Fig 45:**
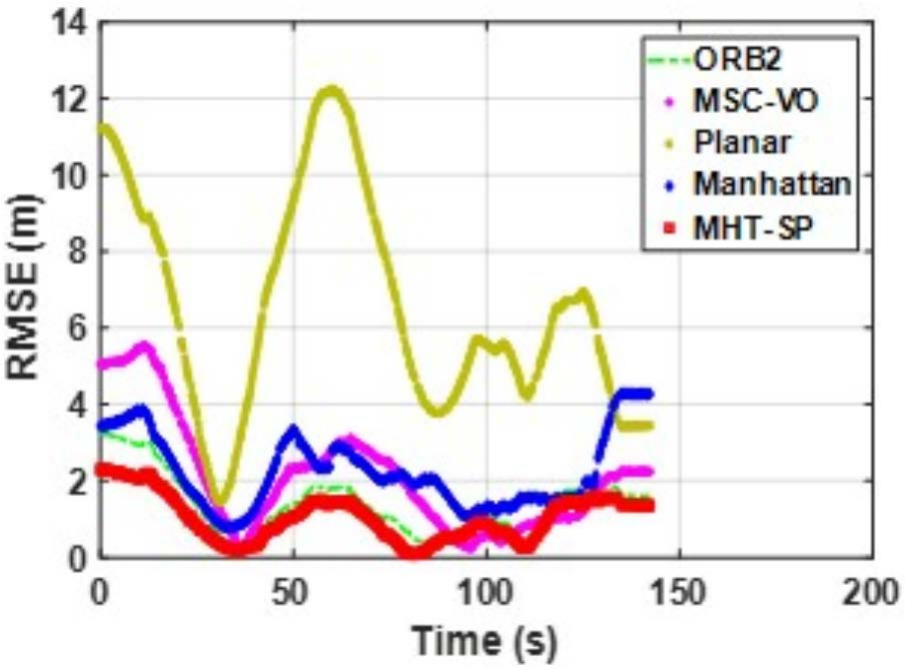
RMSE comparison of Data_2-2 based on the actual scene measured by ZED2i camera.

**Fig 46:**
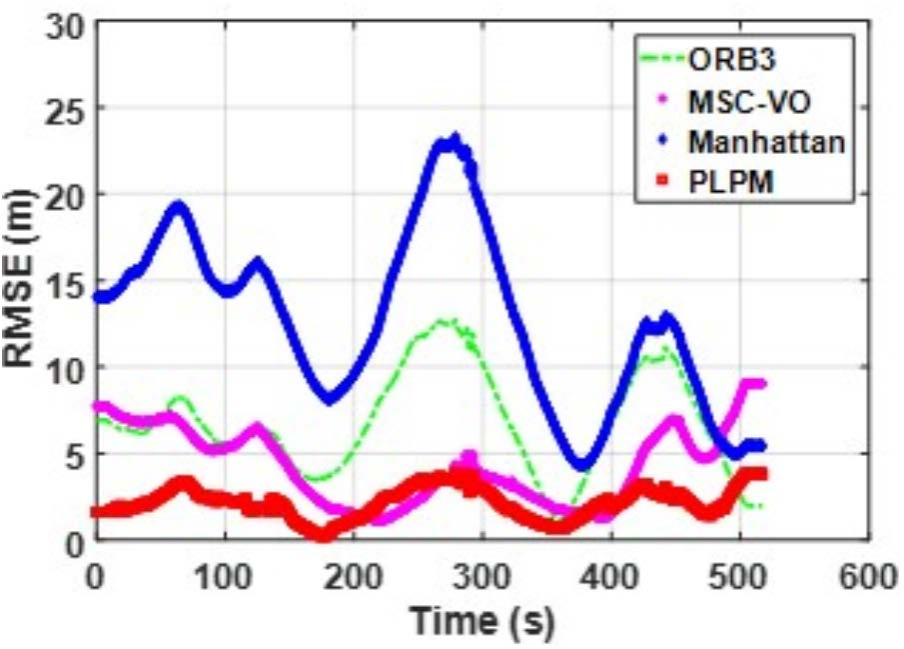
RMSE comparison of Data_2-3 based on the actual scene measured by ZED2i camera.

Figs 41–43 show the trajectory comparisons, and Figs 44–46 show the corresponding RMSE results, for selected cases in the long corridor and underground garage scenes. The detailed comparison results are shown in Table 4.

**Table 4:** Evaluation results of translation ATE RMSE (unit: m) on long corridor scene and the underground garage scene. Bold numbers represent the best performances.

Type	Dataset	Distance (m)	ORB-SLAM3	Manhattan-SLAM	Planar-SLAM	MSC-VO	PLP-SLAM	PLPM-SLAM	Increase (%)
Scene 1 (Long corridor)	Data_1-1	26.5	0.1150	0.2801	5.5996	0.3951	0.0956	**0.0693**	**39.74**
	Data_1-2	26.9	0.5886	0.8636	2.6594	0.4974	0.4438	**0.4032**	**31.50**
	Data_1-3	46.4	1.4650	10.6689	11.2587	4.2955	0.3012	**0.1148**	**92.16**
Scene 2 (Underground parking lot)	Data_2-1	61.1	1.6295	2.4229	7.2819	2.5906	1.4457	**1.2209**	**25.08**
	Data_2-2	54.8	1.4802	2.2011	7.0624	1.2348	1.3346	**1.1246**	**24.02**
	Data_2-3	240.2	7.3907	13.6787	*×*	4.8358	3.6521	**2.3096**	**68.75**

From the comparison in the Table 4, it can be observed that among the various methods compared, Planar-SLAM performs the worst in terms of both performance and stability in real-world scenarios, regardless of long corridor or underground parking lot, because it cannot be applied to non-MF environments. When the duration and distance of the scene are short, ORB-SLAM3 can maintain relatively stable accuracy. However, as the distance and time increase, the accuracy of ORB-SLAM3 gradually decreases due to error accumulation.

MSC-VO primarily relies on line features for MF extraction. Overall, it exhibits relatively stable performance, particularly in the Data_1–2 and Data_2–2 sequences. However, in Data_2–1, which includes dynamic elements in certain frames, the performance of all compared methods—except for the proposed algorithm—deteriorates, even though the sequence length in Data_2–2 is comparatively shorter. While MSC-VO, Manhattan-SLAM, and the proposed method are all capable of handling both structured and unstructured environments, MSC-VO is limited to scenes containing a single dominant MF. In cases where multiple dominant directions exist, the structural constraint module in MSC-VO becomes ineffective. For example, in Data_1–1 and Data_1–2, since the dominant MF direction remains consistent, MSC-VO performs relatively well. However, Manhattan-SLAM, due to the absence of a Local Bundle Adjustment (BA) module, suffers from higher RMSE in larger-scale scenes—particularly in frames with sparse orthogonal plane feature observations. In the three datasets from Scene 2, representing an underground parking garage, the proposed algorithm achieves the best performance, though RMSE still exceeds 1 meter. Scene 2 is characterized by dim lighting, irregular structural geometry, and numerous parked vehicles, which contribute to disorganized and fragmented line features. While some planes are detected, few satisfy orthogonality constraints, complicating accurate MF extraction.

Both Manhattan-SLAM and Planar-SLAM utilize point cloud clustering to extract plane features and employ angular and distance constraints between plane normals for matching. However, these methods struggle in irregular environments where reliable and consistent plane features cannot be extracted—especially across widely separated frames. In such cases, error accumulation makes these coarse constraints insufficient for accurate plane association. To mitigate this, the proposed algorithm augments point cloud clustering with coplanar line features to construct additional plane hypotheses, thereby introducing more geometric constraints into the backend optimization. While Manhattan-SLAM typically fails under such irregular conditions, the proposed model maintains stable performance. By comparing results from public datasets with those from real-world experiments described in this section, it becomes evident that Manhattan-SLAM and Planar-SLAM are highly dependent on scene-specific structural characteristics. Planar-SLAM, in particular, performs well in environments with strong planar geometry (e.g., living_room_traj1_frei) but exhibits reduced robustness under more diverse real-world conditions. In contrast, ORB-SLAM3 and MSC-VO, although not always achieving the best accuracy, maintain relatively stable performance with minimal error fluctuations and no observed tracking failures.

**Fig 47:**
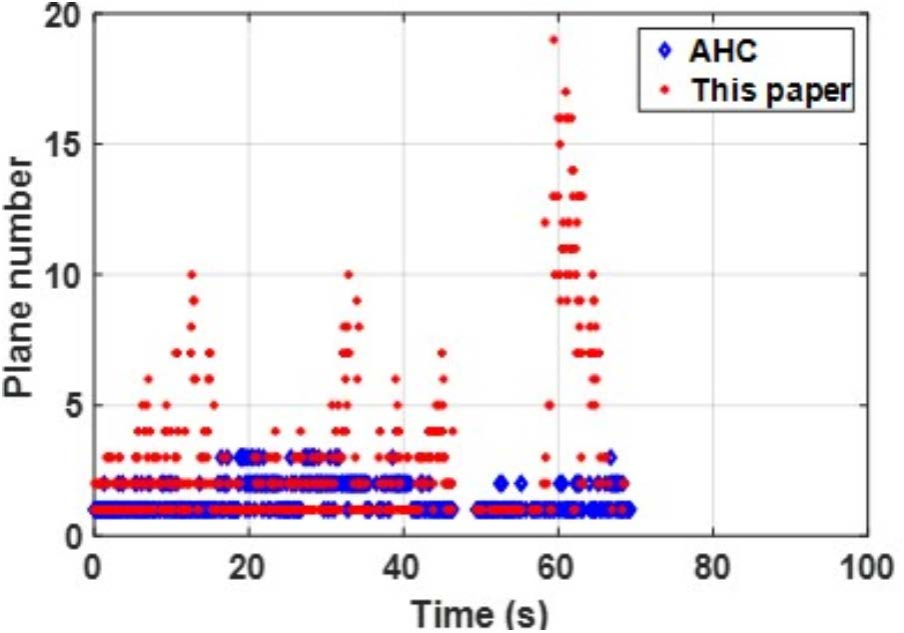
Comparison of the number of extracted plane features in scenes Data_1-1.

**Fig 48:**
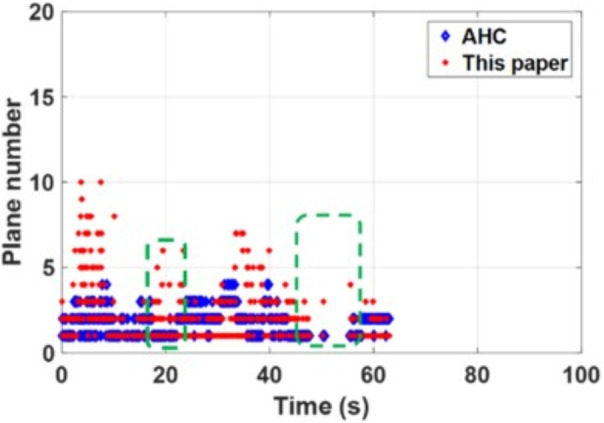
Comparison of the number of extracted plane features in scenes Data_1-2.

**Fig 49:**
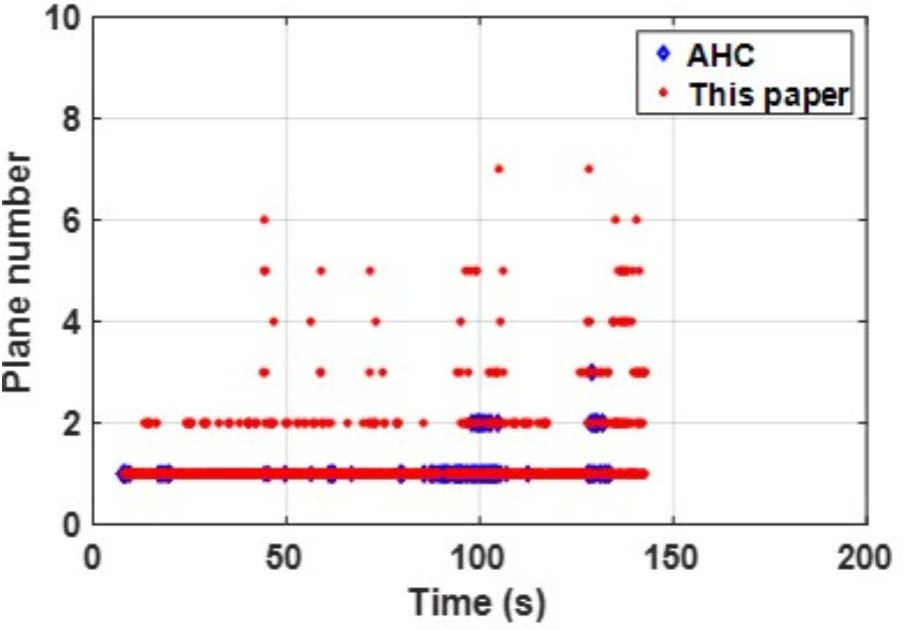
Comparison of the number of extracted plane features in scenes Data_2-1.

**Fig 50:**
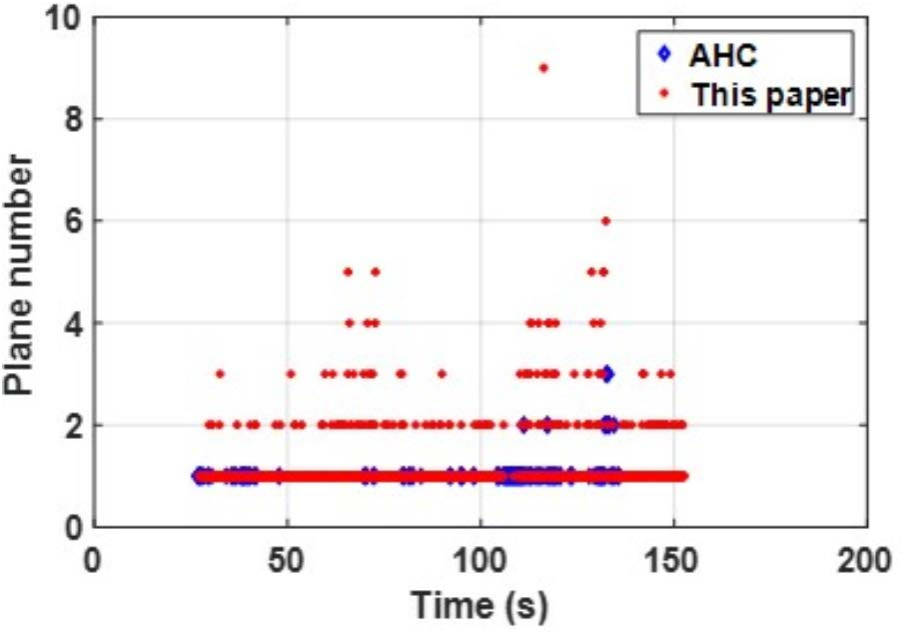
Comparison of the number of extracted plane features in scenes Data_2-2.

In summary, PLPM-SLAM consistently achieves the highest accuracy and robustness across both benchmark and real-world datasets, with especially pronounced advantages in real-world experimental scenarios. The number of plane features extracted by the AHC model and the proposed algorithm is compared in Figs 47–48 for Scene 1 and in Figs 49–50 for Scene 2. It is evident that the proposed method extracts a significantly larger number of plane features, particularly in complex regions such as corners (highlighted areas). Compared with the AHC model, the proposed approach provides more abundant planar constraints, thereby enhancing backend pose optimization. By analyzing Figs 47–50, it is also observed that both the AHC model and the proposed method extract more plane features in long corridors (Scene 1) than in underground parking lots (Scene 2), due to better-defined structural regularities in corridor environments.

## 5 Conclusion

This paper introduces PLPM-SLAM, an RGB-D SLAM system that integrates virtual-plane–assisted MF constraints with point-line-plane joint optimization to mitigate localization degradation and global drift in complex indoor environments. By incorporating both homogeneous and heterogeneous geometric relationships, the proposed system significantly enhances tracking robustness and pose estimation accuracy. Extensive evaluations on public benchmark datasets (TUM, ICL-NUIM) and real-world indoor scenes captured using a ZED2i RGB-D camera demonstrate that PLPM-SLAM consistently outperforms baseline methods, particularly in environments characterized by structural regularity or low texture.

Nevertheless, in highly cluttered or weakly structured scenes, the system’s performance may be constrained by limited plane feature availability. Future research will focus on leveraging deep learning–based structural plane detection and robust feature matching strategies to improve adaptability and accuracy in such challenging environments.
